# Supplementary material for: Gabapentinoid consumption in 65 countries and regions from 2008 to 2018: a longitudinal trend study
Source: Nat Commun. 2023 Aug 17;14:5005. doi: 10.1038/s41467-023-40637-8 (PMC10435503; doi:10.1038/s41467-023-40637-8)
Supplement: Supplementary file 1 — Supplementary Information [file 41467_2023_40637_MOESM1_ESM.docx]

**Supplementary Information**

**Gabapentinoid consumption in 65 countries and regions from 2008 to 2018 a longitudinal trend study**

Adrienne Y.L. Chan^1,2,3,4#^, Andrew S.C. Yuen^4#^, Daniel H.T. Tsai^5,6^, Wallis C.Y. Lau^2,3,4,7^, Yogini H. Jani^4,7^, Yingfen Hsia^5,8^, David P.J. Osborn^9^, Joseph F. Hayes^9^, Frank M.C. Besag^4,10^, Edward C.C. Lai^6^, Li Wei^4,7^, Katja Taxis^1^, Ian C.K. Wong^2,3,4,7,11^*, Kenneth K.C. Man^2,3,4,7^*

^1^ Groningen Research Institute of Pharmacy, Unit of Pharmacotherapy Epidemiology and Economics, University of Groningen, 72 9700 Groningen, The Netherlands

^2^ Laboratory of Data Discovery for Health (D^2^4H), Hong Kong Science Park, Hong Kong SAR, China

^3^ Centre for Safe Medication Practice and Research, Department of Pharmacology and Pharmacy, Li Ka Shing Faculty of Medicine, The University of Hong Kong, Hong Kong SAR, China

^4^ Research Department of Practice and Policy, School of Pharmacy, University College London, London, WC1H 9JP, United Kingdom

^5^ Centre for Neonatal and Paediatric Infection, St George’s University of London, SW17 0RE, United Kingdom

^6^ Institute of Clinical Pharmacy and Pharmaceutical Sciences, College of Medicine, National Cheng Kung University, Tainan, Taiwan.

^7^ Centre for Medicines Optimisation Research and Education, University College London Hospitals NHS Foundation Trust, London, NW1 2PG, United Kingdom

^8^ School of Pharmacy, Queen's University, Belfast, BT9 7BL, United Kingdom

^9^ Division of Psychiatry, University College London, London, W1T 7BN, United Kingdom

^10^ East London Foundation NHS Trust, Bedfordshire, MK40 3JT, United Kingdom

^11^Aston School of Pharmacy, Aston University, Birmingham, B4 7ET, United Kingdom

^#^ The first two authors have contributed equally to the manuscript

*Co-corresponding authors. Emails for correspondence: [wongick@hku.hk](mailto:wongick@hku.hk) (I.C.K.W.), [kenneth.man@ucl.ac.uk](mailto:kenneth.man@ucl.ac.uk) (K.K.C.M.)

# **Supplementary Tables**

# **Supplementary Table 1. Licensed indications of use of Gabapentin, Pregabalin and Gabapentin Enacarbil (as of 31^st^ May, 2023)**

| **Country/ Region** | **Drug** | **Approved indication(s) of use/ Remarks** |
| --- | --- | --- |
| Canada | Gabapentin^1^ | Adjunctive therapy for the management of patients with epilepsy who are not satisfactorily controlled by conventional therapy |
|  | Pregabalin^1^ | 1. Neuropathic pain associated with diabetic peripheral neuropathy 2. Neuropathic pain associated with postherpetic neuralgia 3. Neuropathic pain associated with spinal cord injury 4. Pain associated with fibromyalgia |
| United States | Gabapentin^2^ | 1. Postherpetic neuralgia 2. Adjunctive therapy in the treatment of partial onset seizures, with and without secondary generalization, in adults and paediatric patients 3 years and older with epilepsy |
|  | Pregabalin^2^ | 1. Neuropathic pain associated with diabetic peripheral neuropathy 2. Postherpetic neuralgia 3. Adjunctive therapy for the treatment of partial-onset seizures in patients 17 years of age and older 4. Fibromyalgia 5. Neuropathic pain associated with spinal cord injury |
|  | Gabapentin Enacarbil^2^ | 1. Moderate-to-severe primary restless legs syndrome in adults 2. Postherpetic neuralgia in adults |
| Countries within European Medicine Agency  (Austria, Belgium, Bulgaria, Croatia, Czech Republic, Estonia, Finland, France, Germany, Greece, Hungary, Ireland, Italy, Latvia, Lithuania, Luxembourg, Netherlands, Norway, Poland, Portugal, Romania, Slovakia, Slovenia, Spain and Sweden) | Gabapentin^3^ | 1. Adjunctive therapy in the treatment of partial seizures with and without secondary generalization in adults and children aged 6 years and above 2. Monotherapy in the treatment of partial seizures with and without secondary generalization in adults and adolescents aged 12 years and above 3. Peripheral neuropathic pain such as painful diabetic neuropathy and post-herpetic neuralgia in adults |
|  | Pregabalin^4^ | 1. Peripheral and central neuropathic pain in adults 2. Adjunctive therapy in adults with partial seizures with or without secondary generalisation 3. Generalised anxiety disorder in adults |
| United Kingdom | Gabapentin^5^ | 1. Adjunctive treatment of focal seizures with or without secondary generalisation 2. Monotherapy for focal seizures with or without secondary generalisation 3. Peripheral neuropathic pain 4. Menopausal symptoms, particularly hot flushes, in women with breast cancer 5. Oscillopsia in multiple sclerosis 6. Spasticity in multiple sclerosis 7. Muscular symptoms in motor neurone disease |
|  | Pregabalin^6^ | 1. Peripheral and central neuropathic pain 2. Adjunctive therapy for focal seizures with or without secondary generalisation 3. Generalised anxiety disorder |
| Australia | Gabapentin^7^ | 1. Partial seizures, including secondarily generalised tonic-clonic seizures, initially as add-on therapy in adults and children age 3 years and above who have not achieved adequate control with standard anti-epileptic drugs 2. Neuropathic pain |
|  | Pregabalin^7^ | 1. Neuropathic pain 2. Adjunctive therapy in adults with partial seizures with or without secondary generalisation |
| New Zealand | Gabapentin^8^ | 1. Partial seizures, including secondarily generalised tonic-clonic seizures, initially as add-on therapy in adults and children age 3 years and above who have not achieved adequate control with standard anti-epileptic drugs 2. Neuropathic pain |
|  | Pregabalin^8^ | 1. Neuropathic pain 2. Adjunctive therapy in adults with partial seizures with or without secondary generalisation |
| China | Gabapentin^9^ | 1. Postherpetic neuralgia 2. Epilepsy: As an adjuvant treatment of partial seizures with or without secondary generalized seizures in adults and children over 12 years old. It can also be used as an adjuvant therapy for partial seizures in children aged 3-12 |
|  | Pregabalin^10^ | 1. Postherpetic neuralgia 2. Fibromyalgia |
| Taiwan | Gabapentin^11^ | 1. Adjuvant therapy for partial seizures in adults and children aged 3 or above 2. Postherpetic neuralgia neuropathic pain |
|  | Pregabalin^11^ | 1. Neuropathic pain caused by diabetic peripheral neuropathy 2. Postherpetic neuralgia 3. Adjuvant treatment of partial epilepsy in adults 4. Fibromyalgia 5. Neuropathic pain caused by spinal cord injury |
| Japan | Gabapentin^12^ | 1. Treatment with concomitant antiepileptic drugs for partial seizures (including secondary generalized seizure) in patients with epilepsy for whom other antiepileptic drugs are not sufficiently effective 2. Moderate to severe restless legs syndrome |
|  | Pregabalin^13^ | 1. Neuropathic pain 2. Pain associated with fibromyalgia |
|  | Gabapentin Enacarbil^12^ | 1. Treatment with concomitant antiepileptic drugs for partial seizures (including secondary generalised seizure) in patients with epilepsy for whom other antiepileptic drugs are not sufficiently effective 2. Moderate to severe restless legs syndrome |
| South Korea | Gabapentin^14^ | 1. Epilepsy 2. Neuropathic pain |
|  | Pregabalin^14^ | 1. Diabetic peripheral neuropathic pain 2. Postherpetic neuralgia 3. Neuropathic pain due to spinal cord injury 4. Complex regional pain Syndrome 5. Cancer neuropathic pain 6. Post spinal surgery syndrome |
| Chile | Gabapentin^15^ | 1. Monotherapy for the treatment of partial seizures with and without secondary generalization in adults and adolescents 12 years of age and older 2. Adjunctive therapy in the treatment of partial seizures with and without secondary generalization in adults and children 3 years of age and older 3. Diabetic neuropathic pain in adults and adolescents 12 years of age and older 4. Post-herpetic neuralgia in adults and adolescents 12 years of age and older. |
|  | Pregabalin^15^ | 1. Neuropathic pain in adults 2. Adjunctive therapy in adults with partial seizures with or without secondary generalization 3. Generalized anxiety disorder 4. Fibromyalgia |
| Puerto Rico | Not found^16^ | Source: Portal del Departamento de Salud |
| Uruguay | Not found^17^ | Source: Ministry of Public Health Online drug information |
| Switzerland | Gabapentin^18^ | 1. Monotherapy in patients 12 years and older with partial-onset seizures with and without secondary generalization 2. Adjunctive therapy in patients 3 years and older with partial seizures with and without secondary generalization 3. Neuropathic pain associated with diabetic neuropathy in adults 4. Neuropathic pain associated with post-herpetic neuralgia adults |
|  | Pregabalin^18^ | 1. Peripheral and central neuropathic pain in adults 2. Add-on therapy for partial onset seizures with or without secondary generalization in adult patients who have had an inadequate response to other antiepileptic drugs 3. Generalized anxiety disorder |
| Kuwait | Not found^19^ | Source: Ministry of health |
| Saudi Arabia | Not found^20^ | Source: Saudi Food and Drug Authority Drugs List |
| United Arab Emirates | Not found^21^ | Source: Ministry of Health and Prevention Registered Medical Product Directory |
| Argentina | Gabapentin^22^ | 1. Adjunctive therapy in the treatment of epileptic patients in adults and children older than 6 years or more 2. Monotherapy in the treatment of partial epilepsy attacks with and without secondary generalizations in adults and adolescents 12 years of age or older 3. Diabetic neuropathic pain in adults 4. Post-herpetic neuralgia in adults |
|  | Pregabalin^22^ | 1. Peripheral and central neuropathic pain in adults 2. Adjunctive therapy in adults with partial seizures with or without secondary generalization 3. Generalized anxiety disorder 4. Fibromyalgia |
| Brazil | Not found^23^ | Source: ANVISA - National Health Surveillance Agency Consultas |
| Colombia | Not found^24^ | Source: Instituto Nacional de Vigilancia de Medicamentos y Alimentos |
| Ecuador | Not found^25^ | Source: Agencia Nacional de Regulación, Control y Vigilancia Sanitaria Medication Consultation |
| Mexico | Not found^26^ | Source: COFEPRIS Listados de Registros Sanitarios de Medicamentos |
| Peru | Not found^27^ | Source: DIGEMID Consulta de Registro Sanitario de Productos Farmacéuticos |
| Venezuela | Not found | Not found (Drug regulatory authority not found) |
| Belarus | Not found^28^ | Source: State Register of Medicinal Products of the Republic of Belarus |
| Russia | Not found^29^ | Source: Ministry of Health of Russian Federation |
| Bosnia and Herzegovina | Not found^30^ | Source: Agency for Medicinal Products and Medical Devices of Bosnia and Herzegovina |
| Serbia | Gabapentin^31^ | 1. Additional therapy for treatment of epilepsy 2. Monotherapy for the treatment of epilepsy in adults and adolescents aged 12 and older 3. Peripheral neuropathic pain, such as diabetic neuropathic pain or post-herpetic neuralgia |
|  | Pregabalin^31^ | 1. Peripheral and central neuropathic pain 2. As a supplement to existing therapy for partial convulsions with or without secondary generalizations in adults if the condition is not under control with existing therapy as a supplement 3. Generalized anxiety disorder |
| Kazakhstan | Gabapentin^32^ | Monotherapy or adjunctive therapy of partial epileptic seizures with or without secondary spread |
|  | Pregabalin^32^ | 1. Peripheral and central neuropathic pain 2. Generalized anxiety disorder 3. Adjunctive therapy in epilepsy with partial seizures with or without secondary generalizations |
| Jordan | Gabapentin^33^ | 1. Adjunctive therapy in the treatment of partial seizures with and without secondary generalization in adults and children aged 6 years and above 2. Monotherapy in the treatment of partial seizures with and without secondary generalization in adults and adolescents aged 12 years and above 3. Peripheral neuropathic pain such as diabetic neuropathy and post-herpetic neuralgia |
|  | Pregabalin^33^ | 1. Peripheral and central neuropathic pain 2. Adjunctive therapy in adults with partial seizures with or without secondary generalisation 3. Fibromyalgia 4. Generalised anxiety disorder |
| Lebanon | Not found^34^ | Source: Lebanon National Drugs Database |
| Türkiye | Not found^35^ | Source: Turkish Medicines and Medical Devices Agency |
| Thailand | Not found^36^ | Source: Food and Drug Administration of Thailand |
| Algeria | Not found^37^ | Source: Ministry of Health, Population and Hospital Reform of Algeria |
| South Africa | Not found^38^ | Source: South African Health Products Regulatory Authority (SAHPRA) |
| Philippines | Not found^39^ | Source: Food and Drug Administration of the Philippines |
| India | Not found^40^ | Source: Central Drugs Standard Control Organization |
| Pakistan | Not found^41^ | Source: Drug Regulatory Authority of Pakistan. Registered drug index |
| Egypt | Not found^42^ | Source: Egyptian Drug Authority |
| Morocco | Not found^43^ | Source: Ministère de la Santé |
| Tunisia | Gabapentin^44^ | 1. Monotherapy treatment (including first-line treatment) in adults and children over 12 years of age, with simple and complex partial epileptic seizures, with or without secondary generalisation 2. Adjunctive therapy in adults and children aged 3 years and over, presenting with simple and complex partial epileptic seizures, with or without secondary generalisation 3. Neuropathic pain |
|  | Pregabalin^44^ | 1. Neuropathic pain 2. Adjunctive therapy for partial epileptic seizures with or without secondary generalisation 3. Generalised anxiety disorder |

Note: Licensed indications of Gabapentin, Pregabalin and Gabapentin enacarbil are identified in 41 countries/ regions as shown above. The above 41 countries/ regions have accounted for over 80% of the total consumption of gabapentinoids in 2018. The information above were retrieved following the hierarchy below:

(1) National drug authority or National Formulary

(2) Approved patient information leaflet or prescribing information uploaded online by the marketing authorisation holder (if licensed indications cannot be found from the first level of the hierarchy)

(3) Reputable third party website that documents licensed indication(s) in the country/ region in interest (if licensed indications cannot be found from the first and second level of the hierarchy)

“Not found” will be shown under the “Drug” column if none of the above sources can provide information regarding approved use of gabapentinoids.

# **Supplementary Table 2. Defined daily dose of different gabapentinoids according to the World Health Organisation Anatomical Therapeutic Chemical Classification System**

| **ATC code** | **Name** | **Defined daily dose** | **Unit** |
| --- | --- | --- | --- |
| N03AX12 | [Gabapentin](https://www.whocc.no/atc_ddd_index/?code=N03AX12) | 1.8 | g |
| N03AX16 | [Pregabalin](https://www.whocc.no/atc_ddd_index/?code=N03AX16&showdescription=yes) | 0.3 | g |
| NA^a^ | [Gabapentin](https://www.whocc.no/atc_ddd_index/?code=N06BA03&showdescription=yes) enacarbil | 0.6 | g |

^a^Defined daily dose was not available from the World Health Organisation Anatomical Therapeutic Chemical Classification System thus was determined by half of the maximum dose (1.2g) as suggested by reputable pharmacy references such as Martindale and UpToDate^45,46^.

# **Supplementary Table 3. Geographical region and income classification of countries/ regions and availability of different gabapentinoids in 2018**

| **Country/ Region** | **Geographical Region** | **Income level** | **Sectors covered** | **Gabapentin** | **Gabapentin enacarbil** | **Pregabalin** |
| --- | --- | --- | --- | --- | --- | --- |
| Canada | Northern America | HIC | Hospital and Retail | Yes | NA | Yes |
| United States | Northern America | HIC | Hospital and Retail | Yes | Yes | Yes |
| Chile | Central and Southern America and the Caribbean | HIC | Retail | Yes | NA | Yes |
| Puerto Rico | Central and Southern America and the Caribbean | HIC | Hospital and Retail | Yes | Yes | Yes |
| Uruguay | Central and Southern America and the Caribbean | HIC | Retail | Yes | NA | Yes |
| Austria | Western Europe | HIC | Hospital and Retail | Yes | NA | Yes |
| Belgium | Western Europe | HIC | Hospital and Retail | Yes | NA | Yes |
| France | Western Europe | HIC | Hospital and Retail | Yes | NA | Yes |
| Germany | Western Europe | HIC | Hospital and Retail | Yes | NA | Yes |
| Luxembourg | Western Europe | HIC | Retail | Yes | NA | Yes |
| Netherlands | Western Europe | HIC | Hospital and Retail | Yes | NA | Yes |
| Switzerland | Western Europe | HIC | Hospital and Retail | Yes | NA | Yes |
| Estonia | Northern Europe | HIC | Retail | Yes | NA | Yes |
| Finland | Northern Europe | HIC | Hospital and Retail | Yes | NA | Yes |
| Ireland | Northern Europe | HIC | Hospital and Retail | Yes | NA | Yes |
| Latvia | Northern Europe | HIC | Hospital and Retail | Yes | NA | Yes |
| Lithuania | Northern Europe | HIC | Hospital and Retail | Yes | NA | Yes |
| Norway | Northern Europe | HIC | Hospital and Retail | Yes | NA | Yes |
| Sweden | Northern Europe | HIC | Hospital and Retail | Yes | NA | Yes |
| United Kingdom | Northern Europe | HIC | Hospital and Retail | Yes | NA | Yes |
| Czech Republic | Eastern Europe | HIC | Hospital and Retail | Yes | NA | Yes |
| Hungary | Eastern Europe | HIC | Hospital and Retail | Yes | NA | Yes |
| Poland | Eastern Europe | HIC | Hospital and Retail | Yes | NA | Yes |
| Slovakia | Eastern Europe | HIC | Hospital and Retail | Yes | NA | Yes |
| Croatia | Southern Europe | HIC | Hospital and Retail | Yes | NA | Yes |
| Greece | Southern Europe | HIC | Retail | Yes | NA | Yes |
| Italy | Southern Europe | HIC | Hospital and Retail | Yes | NA | Yes |
| Portugal | Southern Europe | HIC | Hospital and Retail | Yes | NA | Yes |
| Slovenia | Southern Europe | HIC | Retail | Yes | NA | Yes |
| Spain | Southern Europe | HIC | Hospital and Retail | Yes | NA | Yes |
| Australia | Australia and New Zealand | HIC | Hospital and Retail | Yes | NA | Yes |
| New Zealand | Australia and New Zealand | HIC | Hospital and Retail | Yes | NA | Yes |
| Japan* | Eastern Asia | HIC | Hospital and Retail | Yes | Yes | Yes |
| South Korea | Eastern Asia | HIC | Retail | Yes | NA | Yes |
| Taiwan | Eastern Asia | HIC | Hospital | Yes | NA | Yes |
| Kuwait | Western Asia | HIC | Retail | Yes | NA | Yes |
| Saudi Arabia | Western Asia | HIC | Retail | Yes | NA | Yes |
| United Arab Emirates | Western Asia | HIC | Retail | Yes | NA | Yes |
| Argentina | Central and Southern America and the Caribbean | UMIC | Retail | Yes | NA | Yes |
| Brazil | Central and Southern America and the Caribbean | UMIC | Retail | Yes | NA | Yes |
| Colombia | Central and Southern America and the Caribbean | UMIC | Retail | Yes | NA | Yes |
| Ecuador | Central and Southern America and the Caribbean | UMIC | Retail | Yes | NA | Yes |
| Mexico | Central and Southern America and the Caribbean | UMIC | Retail | Yes | NA | Yes |
| Peru | Central and Southern America and the Caribbean | UMIC | Retail | Yes | NA | Yes |
| Venezuela | Central and Southern America and the Caribbean | UMIC | Retail | Yes | NA | Yes |
| Belarus | Eastern Europe | UMIC | Hospital and Retail | Yes | NA | Yes |
| Bulgaria | Eastern Europe | UMIC | Hospital and Retail | Yes | NA | Yes |
| Romania | Eastern Europe | UMIC | Hospital and Retail | Yes | NA | Yes |
| Russia | Eastern Europe | UMIC | Hospital and Retail | Yes | NA | Yes |
| Bosnia and Herzegovina | Southern Europe | UMIC | Retail | Yes | NA | Yes |
| Serbia | Southern Europe | UMIC | Retail | Yes | NA | Yes |
| China | Eastern Asia | UMIC | Hospital and Retail | Yes | NA | Yes |
| Kazakhstan | Central Asia | UMIC | Hospital and Retail | Yes | NA | Yes |
| Jordan | Western Asia | UMIC | Retail | Yes | NA | Yes |
| Lebanon | Western Asia | UMIC | Hospital and Retail | Yes | NA | Yes |
| Türkiye | Western Asia | UMIC | Hospital and Retail | Yes | NA | Yes |
| Thailand | South-eastern Asia | UMIC | Hospital | Yes | NA | Yes |
| Algeria | Northern Africa | UMIC | Retail | Yes | NA | Yes |
| South Africa | Southern Africa | UMIC | Retail | Yes | NA | Yes |
| Philippines | South-eastern Asia | LMIC | Hospital and Retail | Yes | NA | Yes |
| India | Southern Asia | LMIC | Retail | Yes | NA | Yes |
| Pakistan | Southern Asia | LMIC | Retail | Yes | NA | Yes |
| Egypt | Northern Africa | LMIC | Retail | Yes | NA | Yes |
| Morocco | Northern Africa | LMIC | Retail | Yes | NA | Yes |
| Tunisia | Northern Africa | LMIC | Hospital and Retail | Yes | NA | Yes |

*According to the MIDAS dataset, out of all 65 studied countries/ regions, only Japan does not have generic versions of Gabapentin and Pregabalin.

Abbreviations: HIC, high income country; UMIC, upper-middle income country; LMIC, lower-middle income country; NA, not applicabl

# **Supplementary Table 4. Results of sensitivity analyses after removing Bosnia And Herzegovina, Kazakhstan, Netherlands, Serbia and Thailand**

|  | **DDD/TID in 2008 (95%CI)^a^** | **DDD/TID in 2018 (95%CI)^a^** | **Average annual percentage change (%, 95%CI)^b^** |
| --- | --- | --- | --- |
| **Multinational** | 4.17 (2.99, 5.81) | 18.26 (13.54, 24.63) | 16.68 (14.97, 18.41) |
| **America (North)** | 52.44 (32.79, 83.86) | 124.62 (95.77, 162.16) | 9.04 (6.82, 11.32) |
| Canada | 41.27 (41.25, 41.28) | 108.95 (108.94, 108.97) | 10.59 (9.70, 11.49) |
| United States | 66.63 (66.63, 66.64) | 142.54 (142.53, 142.55) | 7.89 (6.82, 8.97) |
| **America (Central and Southern) and the Caribbean** | 2.33 (0.90, 6.02) | 7.86 (3.51, 17.58) | 12.92 (9.55, 16.39) |
| Argentina | 2.11 (2.11, 2.11) | 13.90 (13.90, 13.91) | 20.53 (14.57, 26.80) |
| Brazil | 0.52 (0.52, 0.52) | 6.32 (6.32, 6.32) | 26.82 (22.90, 30.86) |
| Chile | 2.11 (2.11, 2.12) | 9.28 (9.27, 9.29) | 17.95 (16.07, 19.85) |
| Colombia | 0.88 (0.87, 0.88) | 1.96 (1.95, 1.96) | 10.48 (7.45, 13.61) |
| Ecuador | 1.95 (1.94, 1.95) | 5.48 (5.47, 5.48) | 10.27 (8.31, 12.28) |
| Mexico | 2.79 (2.79, 2.79) | 4.13 (4.12, 4.13) | 3.84 (1.93, 5.78) |
| Peru | 0.50 (0.50, 0.50) | 2.56 (2.56, 2.56) | 19.06 (16.71, 21.47) |
| Puerto Rico | 50.09 (50.05, 50.13) | 151.23 (151.16, 151.30) | 12.33 (10.51, 14.17) |
| Uruguay | 2.54 (2.53, 2.55) | 15.40 (15.38, 15.42) | 19.16 (12.91, 25.75) |
| Venezuela | 6.79 (6.79, 6.80) | 4.18 (4.18, 4.19) | -3.70 (-11.98, 5.36) |
| **Europe (West)** | 23.77 (19.12, 29.55) | 52.81 (49.32, 56.54) | 8.31 (6.64, 10.00) |
| Austria | 21.98 (21.96, 21.99) | 60.50 (60.48, 60.53) | 10.41 (8.93, 11.91) |
| Belgium | 13.01 (13.00, 13.02) | 56.26 (56.24, 56.29) | 14.62 (12.07, 17.23) |
| France | 35.47 (35.46, 35.47) | 60.18 (60.17, 60.19) | 5.39 (4.72, 6.07) |
| Germany | 27.85 (27.84, 27.85) | 56.64 (56.63, 56.64) | 6.93 (5.83, 8.04) |
| Luxembourg | 32.51 (32.43, 32.60) | 44.24 (44.15, 44.33) | 3.11 (2.37, 3.87) |
| Netherlands | Removed | Removed | Removed |
| Switzerland | 19.62 (19.61, 19.64) | 42.26 (42.24, 42.28) | 8.11 (7.03, 9.20) |
| **Europe (North)** | 13.77 (11.03, 17.21) | 54.66 (38.59, 77.43) | 14.78 (12.34, 17.27) |
| Estonia | 2.02 (2.01, 2.03) | 30.00 (29.95, 30.05) | 29.97 (27.75, 32.23) |
| Finland | 43.56 (43.53, 43.59) | 82.83 (82.79, 82.87) | 5.73 (4.61, 6.87) |
| Ireland | 28.73 (28.70, 28.75) | 85.54 (85.50, 85.58) | 13.75 (10.33, 17.27) |
| Latvia | 4.16 (4.15, 4.18) | 26.39 (26.36, 26.43) | 21.02 (19.53, 22.53) |
| Lithuania | 3.47 (3.46, 3.48) | 20.12 (20.09, 20.15) | 19.49 (16.96, 22.07) |
| Norway | 34.30 (34.27, 34.33) | 66.32 (66.28, 66.36) | 6.82 (6.07, 7.57) |
| Sweden | 33.98 (33.96, 34.00) | 76.67 (76.64, 76.70) | 7.99 (7.35, 8.62) |
| United Kingdom | 30.46 (30.45, 30.46) | 138.88 (138.86, 138.89) | 16.99 (14.83, 19.19) |
| **Europe (South)** | 16.40 (10.55, 25.48) | 37.51 (24.42, 57.62) | 8.63 (6.82, 10.47) |
| Bosnia And Herzegovina | Removed | Removed | Removed |
| Croatia | 3.16 (3.15, 3.17) | 13.82 (13.80, 13.83) | 15.53 (12.92, 18.21) |
| Greece | 22.48 (22.47, 22.50) | 46.34 (46.32, 46.36) | 6.44 (5.24, 7.65) |
| Italy | 14.92 (14.91, 14.92) | 28.82 (28.81, 28.82) | 6.64 (5.64, 7.65) |
| Portugal | 31.22 (31.21, 31.24) | 51.52 (51.50, 51.55) | 5.10 (3.82, 6.40) |
| Serbia | Removed | Removed | Removed |
| Slovenia | 15.31 (15.28, 15.34) | 40.00 (39.95, 40.04) | 10.06 (9.17, 10.95) |
| Spain | 38.35 (38.34, 38.36) | 73.28 (73.27, 73.29) | 6.17 (4.91, 7.44) |
| **Europe (East)** | 1.76 (0.70, 4.42) | 14.88 (7.27, 30.44) | 23.77 (17.06, 30.86) |
| Belarus | 0.06 (0.06, 0.06) | 1.87 (1.86, 1.87) | 35.74 (30.09, 41.63) |
| Bulgaria | 1.05 (1.05, 1.06) | 13.77 (13.76, 13.79) | 24.48 (18.37, 30.90) |
| Czech Republic | 11.42 (11.41, 11.43) | 60.55 (60.52, 60.57) | 17.84 (16.42, 19.27) |
| Hungary | 7.21 (7.20, 7.22) | 21.51 (21.49, 21.52) | 11.43 (9.05, 13.87) |
| Poland | 1.51 (1.51, 1.51) | 16.8 (16.79, 16.80) | 26.34 (19.72, 33.32) |
| Romania | 3.11 (3.10, 3.11) | 16.37 (16.36, 16.37) | 15.43 (12.31, 18.64) |
| Russia | 0.29 (0.29, 0.29) | 5.05 (5.05, 5.05) | 32.16 (11.78, 56.24) |
| Slovakia | 14.11 (14.10, 14.13) | 51.63 (51.59, 51.66) | 12.87 (9.87, 15.94) |
| **Oceania** | 11.27 (8.69, 14.61) | 68.88 (37.14, 127.72) | 19.89 (13.70, 26.43) |
| Australia | 12.86 (12.86, 12.87) | 94.38 (94.36, 94.40) | 26.67 (21.30, 32.27) |
| New Zealand | 9.87 (9.85, 9.88) | 50.26 (50.23, 50.29) | 16.61 (15.77, 17.45) |
| **Asia (East)** | 0.63 (0.10, 4.18) | 7.74 (1.31, 45.89) | 28.51 (18.86, 38.94) |
| China | 0.01 (0.01, 0.01) | 0.44 (0.44, 0.44) | 45.23 (36.07, 55.00) |
| Japan | 1.02 (1.01, 1.02) | 41.32 (41.32, 41.33) | 45.79 (28.56, 65.32) |
| South Korea | 9.48 (9.47, 9.48) | 30.99 (30.99, 31.00) | 12.12 (11.07, 13.17) |
| Taiwan | 1.75 (1.75, 1.75) | 6.41 (6.41, 6.42) | 15.08 (13.02, 17.18) |
| **Asia (Central)** | Removed | Removed | Removed |
| Kazakhstan | Removed | Removed | Removed |
| **Asia (West)** | 3.12 (1.38, 7.06) | 15.73 (6.40, 38.69) | 17.57 (10.53, 25.06) |
| Jordan | 1.27 (1.26, 1.27) | 6.51 (6.50, 6.52) | 24.09 (17.99, 30.51) |
| Kuwait | 1.18 (1.17, 1.18) | 32.25 (32.22, 32.28) | 40.64 (34.36, 47.22) |
| Lebanon | 4.02 (4.01, 4.03) | 15.69 (15.67, 15.70) | 13.02 (10.93, 15.15) |
| Saudi Arabia | 3.62 (3.61, 3.62) | 11.36 (11.35, 11.36) | 16.57 (11.01, 22.40) |
| Türkiye | 13.87 (13.86, 13.87) | 60.98 (60.97, 60.99) | 15.81 (13.63, 18.04) |
| United Arab Emirates | 3.10 (3.09, 3.11) | 6.64 (6.63, 6.65) | 6.55 (-0.91, 14.57) |
| **Asia (South-east)** | 0.53 (0.53, 0.53) | 1.57 (1.57, 1.58) | 10.66 (9.39, 11.94) |
| Philippines | 0.53 (0.53, 0.53) | 1.57 (1.57, 1.58) | 10.40 (9.50, 11.40) |
| Thailand | Removed | Removed | Removed |
| **Asia (South)** | 1.06 (0.90, 1.25) | 4.53 (3.54, 5.78) | 15.56 (12.19, 19.03) |
| India | 1.15 (1.15, 1.15) | 5.13 (5.13, 5.13) | 14.83 (12.63, 17.08) |
| Pakistan | 0.97 (0.97, 0.97) | 3.99 (3.99, 3.99) | 13.21 (11.07, 15.39) |
| **Africa (North)** | 0.68 (0.41, 1.14) | 14.77 (8.49, 25.70) | 35.91 (26.17, 46.41) |
| Algeria | 0.71 (0.71, 0.71) | 29.44 (29.43, 29.44) | 36.74 (27.43, 46.72) |
| Egypt | 1.06 (1.06, 1.07) | 39.54 (39.54, 39.55) | 39.14 (35.14, 43.27) |
| Morocco | 0.24 (0.23, 0.24) | 2.01 (2.01, 2.01) | 20.52 (17.94, 23.16) |
| Tunisia | 1.22 (1.22, 1.23) | 20.33 (20.31, 20.34) | 24.68 (18.90, 30.74) |
| **Africa (South)** | 1.16 (1.16, 1.16) | 3.74 (3.74, 3.74) | 12.42 (6.35, 18.85) |
| South Africa | 1.16 (1.16, 1.16) | 3.74 (3.74, 3.74) | 10.88 (8.73, 13.07) |

^a^Worldwide and regional estimates with 95% CI were calculated by pooling the estimates using meta-analysis (random-effects model).

^b^The average annual change is calculated using a linear regression model, with log-transformed consumption in DDD/TID as the dependent variable and year as the independent variable. The average annual change was expressed as average annual percentage change, calculated by [exp(the coefficient of the year variable) – 1] x 100%. The multinational and regional trend changes were estimated using linear mixed models, controlling for within-country correlations and assuming the correlations between years were autocorrelated.

Abbreviations: CI, confidence interval; DDD/TID, Defined Daily Dose per 10,000 inhabitants per day

**Supplementary Table 5. Results of sensitivity analyses using only retail sales data**

|  | **DDD/TID in 2008 (95%CI)^a^** | **DDD/TID in 2018 (95%CI)^a^** | **Average annual percentage change (%, 95%CI)^b^** |
| --- | --- | --- | --- |
| **Multinational** | 4.47 (3.23, 6.19) | 17.73 (13.31, 23.62) | 17.49 (15.65, 19.37) |
| **America (North)** | 47.61 (32.68, 69.37) | 115.47 (98.22, 135.74) | 9.27 (7.07, 11.51) |
| Canada | 39.30 (39.29, 39.31) | 106.32 (106.30, 106.34) | 10.83 (9.94, 11.72) |
| United States | 57.69 (57.69, 57.70) | 125.40 (125.40, 125.41) | 8.28 (7.18, 9.38) |
| **America (Central and Southern) and the Caribbean** | 2.32 (0.92, 5.84) | 7.84 (3.55, 17.33) | 12.96 (9.58, 16.44) |
| Argentina | 2.11 (2.11, 2.11) | 13.90 (13.90, 13.91) | 20.53 (14.57, 26.80) |
| Brazil | 0.52 (0.52, 0.52) | 6.32 (6.32, 6.32) | 26.82 (22.90, 30.86) |
| Chile | 2.11 (2.11, 2.12) | 9.28 (9.27, 9.29) | 17.95 (16.07, 19.85) |
| Colombia | 0.88 (0.87, 0.88) | 1.96 (1.95, 1.96) | 10.48 (7.45, 13.61) |
| Ecuador | 1.95 (1.94, 1.95) | 5.48 (5.47, 5.48) | 10.27 (8.31, 12.28) |
| Mexico | 2.79 (2.79, 2.79) | 4.13 (4.12, 4.13) | 3.84 (1.93, 5.78) |
| Peru | 0.50 (0.50, 0.50) | 2.56 (2.56, 2.56) | 19.06 (16.71, 21.47) |
| Puerto Rico | 47.19 (47.16, 47.23) | 147.32 (147.25, 147.39) | 12.74 (10.85, 14.66) |
| Uruguay | 2.54 (2.53, 2.55) | 15.40 (15.38, 15.42) | 19.16 (12.91, 25.75) |
| Venezuela | 6.79 (6.79, 6.80) | 4.18 (4.18, 4.19) | -3.70 (-11.98, 5.36) |
| **Europe (West)** | 22.65 (18.12, 28.32) | 48.98 (44.38, 54.07) | 8.33 (6.81, 9.88) |
| Austria | 20.53 (20.52, 20.55) | 57.99 (57.96, 58.01) | 10.68 (9.12, 12.26) |
| Belgium | 12.57 (12.55, 12.58) | 54.51 (54.48, 54.53) | 14.70 (12.16, 17.30) |
| France | 33.97 (33.96, 33.98) | 58.13 (58.12, 58.14) | 5.50 (4.78, 6.22) |
| Germany | 26.37 (26.36, 26.37) | 53.90 (53.89, 53.91) | 7.01 (5.88, 8.15) |
| Luxembourg | 32.51 (32.43, 32.60) | 44.24 (44.15, 44.33) | 3.11 (2.37, 3.87) |
| Netherlands | 0.00 (0.00, 0.00) | 39.52 (39.51, 39.54) | 7.94 (6.72, 9.18) |
| Switzerland | 17.98 (17.96, 17.99) | 39.07 (39.05, 39.10) | 8.28 (7.21, 9.35) |
| **Europe (North)** | 13.31 (10.68, 16.59) | 53.39 (37.71, 75.58) | 14.90 (12.47, 17.38) |
| Estonia | 2.02 (2.01, 2.03) | 30.00 (29.95, 30.05) | 29.97 (27.75, 32.23) |
| Finland | 40.00 (39.98, 40.03) | 79.96 (79.92, 80.00) | 6.23 (5.05, 7.43) |
| Ireland | 27.23 (27.20, 27.25) | 82.66 (82.62, 82.71) | 14.00 (10.49, 17.63) |
| Latvia | 4.16 (4.15, 4.18) | 25.70 (25.66, 25.74) | 20.45 (19.16, 21.75) |
| Lithuania | 3.44 (3.43, 3.45) | 19.78 (19.75, 19.81) | 19.37 (16.83, 21.96) |
| Norway | 33.15 (33.12, 33.17) | 65.23 (65.19, 65.27) | 7.06 (6.38, 7.74) |
| Sweden | 32.75 (32.73, 32.77) | 74.43 (74.40, 74.45) | 8.12 (7.49, 8.76) |
| United Kingdom | 28.78 (28.78, 28.79) | 134.83 (134.82, 134.85) | 17.30 (15.09, 19.56) |
| **Europe (South)** | 16.01 (10.08, 25.43) | 26.56 (17.50, 40.31) | 14.88 (10.89, 19.02) |
| Bosnia And Herzegovina | 0.00 (0.00, 0.00) | 5.33 (5.32, 5.35) | 35.56 (27.42, 44.22) |
| Croatia | 3.04 (3.03, 3.05) | 13.36 (13.35, 13.38) | 15.36 (12.76, 18.02) |
| Greece | 22.48 (22.47, 22.50) | 46.34 (46.32, 46.36) | 6.44 (5.24, 7.65) |
| Italy | 13.69 (13.68, 13.69) | 26.08 (26.07, 26.09) | 6.50 (5.61, 7.39) |
| Portugal | 31.22 (31.21, 31.24) | 51.17 (51.14, 51.19) | 5.04 (3.77, 6.32) |
| Serbia | 0.00 (0.00, 0.00) | 19.56 (19.54, 19.57) | 57.97 (48.80, 67.70) |
| Slovenia | 15.31 (15.28, 15.34) | 40.00 (39.95, 40.04) | 10.06 (9.17, 10.95) |
| Spain | 37.66 (37.65, 37.67) | 71.76 (71.75, 71.77) | 6.13 (4.87, 7.40) |
| **Europe (East)** | 1.61 (0.66, 3.90) | 14.16 (7.31, 27.41) | 24.30 (17.41, 31.60) |
| Belarus | 0.05 (0.05, 0.05) | 1.79 (1.78, 1.79) | 36.30 (30.36, 42.52) |
| Bulgaria | 0.99 (0.99, 0.99) | 13.62 (13.61, 13.64) | 25.18 (18.96, 31.73) |
| Czech Republic | 7.86 (7.85, 7.87) | 46.66 (46.64, 46.68) | 18.91 (16.95, 20.90) |
| Hungary | 7.08 (7.08, 7.09) | 21.40 (21.38, 21.42) | 11.54 (9.09, 14.03) |
| Poland | 1.50 (1.50, 1.50) | 16.39 (16.39, 16.40) | 26.14 (19.53, 33.12) |
| Romania | 3.02 (3.02, 3.03) | 16.07 (16.06, 16.08) | 15.42 (12.20, 18.73) |
| Russia | 0.26 (0.26, 0.26) | 4.93 (4.93, 4.93) | 32.77 (11.75, 57.74) |
| Slovakia | 13.74 (13.73, 13.76) | 51.17 (51.14, 51.20) | 13.03 (10.01, 16.13) |
| **Oceania** | 9.01 (7.99, 10.16) | 66.86 (37.05, 120.67) | 22.41 (14.68, 30.66) |
| Australia | 8.47 (8.47, 8.48) | 90.37 (90.35, 90.39) | 32.92 (25.38, 40.92) |
| New Zealand | 9.58 (9.57, 9.60) | 49.47 (49.44, 49.51) | 16.76 (15.92, 17.61) |
| **Asia (East)** | 2.20 (0.13, 38.47) | 3.90 (0.99, 15.38) | 38.01 (17.87, 61.59) |
| China | 0.00 (0.00, 0.00) | 0.07 (0.07, 0.07) | 53.22 (27.06, 84.77) |
| Japan | 0.51 (0.51, 0.51) | 28.60 (28.60, 28.61) | 50.24 (30.70, 72.70) |
| South Korea | 9.48 (9.47, 9.48) | 30.99 (30.99, 31.00) | 12.12 (11.07, 13.17) |
| *Taiwan* | No retail data available | No retail data available | No retail data available |
| **Asia (Central)** | 0.00 (0.00, 0.00) | 1.01 (1.01, 1.02) | 19.90 (-0.54, 44.54) |
| Kazakhstan | 0.00 (0.00, 0.00) | 1.01 (1.01, 1.02) | 17.84 (10.84, 25.29) |
| **Asia (West)** | 3.12 (1.38, 7.03) | 15.72 (6.40, 38.60) | 17.58 (10.54, 25.06) |
| Jordan | 1.27 (1.26, 1.27) | 6.51 (6.50, 6.52) | 24.09 (17.99, 30.51) |
| Kuwait | 1.18 (1.17, 1.18) | 32.25 (32.22, 32.28) | 40.64 (34.36, 47.22) |
| Lebanon | 4.02 (4.01, 4.03) | 15.69 (15.67, 15.70) | 13.02 (10.93, 15.15) |
| Saudi Arabia | 3.62 (3.61, 3.62) | 11.36 (11.35, 11.36) | 16.57 (11.01, 22.40) |
| Türkiye | 13.79 (13.79, 13.79) | 60.80 (60.79, 60.81) | 15.83 (13.66, 18.05) |
| United Arab Emirates | 3.10 (3.09, 3.11) | 6.64 (6.63, 6.65) | 6.55 (-0.91, 14.57) |
| **Asia (South-east)** | 0.51 (0.51, 0.51) | 1.53 (1.53, 1.53) | 10.71 (9.41, 12.03) |
| Philippines | 0.51 (0.51, 0.51) | 1.53 (1.53, 1.53) | 10.50 (9.51, 11.49) |
| *Thailand* | No retail data available | No retail data available | No retail data available |
| **Asia (South)** | 1.06 (0.90, 1.25) | 4.53 (3.54, 5.78) | 15.56 (12.19, 19.03) |
| India | 1.15 (1.15, 1.15) | 5.13 (5.13, 5.13) | 14.83 (12.63, 17.08) |
| Pakistan | 0.97 (0.97, 0.97) | 3.99 (3.99, 3.99) | 13.21 (11.07, 15.39) |
| **Africa (North)** | 0.68 (0.41, 1.14) | 14.73 (8.46, 25.66) | 35.87 (26.11, 46.39) |
| Algeria | 0.71 (0.71, 0.71) | 29.44 (29.43, 29.44) | 36.74 (27.43, 46.72) |
| Egypt | 1.06 (1.06, 1.07) | 39.54 (39.54, 39.55) | 39.14 (35.14, 43.27) |
| Morocco | 0.24 (0.23, 0.24) | 2.01 (2.01, 2.01) | 20.52 (17.94, 23.16) |
| Tunisia | 1.22 (1.22, 1.23) | 20.13 (20.12, 20.15) | 24.48 (18.61, 30.64) |
| **Africa (South)** | 1.16 (1.16, 1.16) | 3.74 (3.74, 3.74) | 12.42 (6.35, 18.85) |
| South Africa | 1.16 (1.16, 1.16) | 3.74 (3.74, 3.74) | 10.88 (8.73, 13.07) |
| **Income groups** |  |  |  |
| High income | 1.06 (0.84, 1.33) | 4.03 (3.27, 4.95) | 14.18 (12.40, 15.99) |
| Upper-middle income | 0.13 (0.07, 0.24) | 0.54 (0.29, 0.99) | 22.31 (17.71, 27.09) |
| Lower-middle income | 0.07 (0.06, 0.10) | 0.61 (0.21, 1.75) | 23.28 (18.49, 28.26) |

^a^ Worldwide and regional estimates with 95% CI were calculated by pooling the estimates using meta-analysis (random-effects model).

^b^ The average annual change is calculated using a linear regression model, with log-transformed consumption in DDD/TID as the dependent variable and year as the independent variable. The average annual change was expressed as average annual percentage change, calculated by [exp(the coefficient of the year variable) – 1] x 100%. The multinational and regional trend changes were estimated using linear mixed models, controlling for within-country correlations and assuming the correlations between years were autocorrelated.

Abbreviations: CI, confidence interval; DDD/TID, Defined Daily Dose per 10,000 inhabitants per day

# **Supplementary Table 6. Multinational, regional, and national levels of gabapentin consumption in 2008 and 2018 and average annual percentage change.**

|  | **DDD/TID in 2008 (95%CI)^a^** | **DDD/TID in 2018 (95%CI)^a^** | **Average annual percentage change (%, 95%CI)^b^** |
| --- | --- | --- | --- |
| **Multinational** | 2.08 (1.45, 3.00) | 4.13 (2.71, 6.30) | 8.70 (6.86, 10.56) |
| **America (North)** | 33.37 (18.77, 59.34) | 75.09 (31.55, 178.71) | 8.45 (5.76, 11.21) |
| Canada | 24.88 (24.87, 24.89) | 48.24 (48.23, 48.26) | 7.52 (6.87, 8.16) |
| United States | 44.76 (44.76, 44.76) | 116.87 (116.87, 116.88) | 9.85 (8.52, 11.19) |
| **America (Central and Southern) and the Caribbean** | 1.13 (0.34, 3.68) | 1.06 (0.17, 6.64) | -0.56 (-6.03, 5.23) |
| Argentina | 1.05 (1.05, 1.05) | 1.26 (1.26, 1.26) | 0.74 (-0.91, 2.42) |
| Brazil | 0.52 (0.52, 0.52) | 1.20 (1.20, 1.20) | 9.09 (6.96, 11.28) |
| Chile | 0.42 (0.42, 0.43) | 0.21 (0.21, 0.22) | -6.13 (-9.17, -2.99) |
| Colombia | 0.34 (0.34, 0.34) | 0.15 (0.15, 0.15) | -8.84 (-11.00, -6.63) |
| Ecuador | 0.73 (0.73, 0.73) | 0.51 (0.51, 0.51) | -4.64 (-7.11, -2.11) |
| Mexico | 0.71 (0.71, 0.71) | 1.00 (1.00, 1.00) | 4.01 (3.26, 4.76) |
| Peru | 0.24 (0.24, 0.24) | 0.70 (0.70, 0.70) | 16.32 (6.46, 27.09) |
| Puerto Rico | 37.46 (37.43, 37.49) | 139.25 (139.18, 139.32) | 14.59 (12.23, 16.99) |
| Uruguay | 1.96 (1.95, 1.96) | 3.05 (3.04, 3.06) | 4.43 (-0.35, 9.45) |
| Venezuela | 4.50 (4.49, 4.50) | 0.26 (0.26, 0.26) | -23.62 (-36.57, -8.03) |
| **Europe (West)** | 8.79 (7.02, 10.99) | 10.14 (8.00, 12.86) | 1.95 (0.19, 3.74) |
| Austria | 14.44 (14.43, 14.45) | 20.63 (20.62, 20.65) | 3.66 (2.01, 5.33) |
| Belgium | 3.49 (3.48, 3.50) | 8.77 (8.76, 8.78) | 12.28 (7.59, 17.17) |
| France | 13.79 (13.79, 13.80) | 13.52 (13.51, 13.52) | 0.34 (-0.67, 1.35) |
| Germany | 12.29 (12.29, 12.30) | 16.75 (16.75, 16.76) | 3.03 (1.92, 4.15) |
| Luxembourg | 8.55 (8.51, 8.59) | 6.43 (6.40, 6.47) | -2.55 (-3.20, -1.89) |
| Netherlands | 0.00 (0.00, 0.00) | 8.13 (8.12, 8.14) | 3.51 (2.76, 4.26) |
| Switzerland | 6.30 (6.29, 6.31) | 5.15 (5.14, 5.16) | -1.96 (-2.86, -1.06) |
| **Europe (North)** | 5.68 (4.16, 7.77) | 22.27 (15.44, 32.12) | 14.63 (11.83, 17.51) |
| Estonia | 0.75 (0.75, 0.76) | 12.83 (12.80, 12.86) | 31.06 (24.74, 37.70) |
| Finland | 9.59 (9.58, 9.61) | 30.97 (30.95, 31.00) | 13.46 (11.08, 15.88) |
| Ireland | 9.19 (9.18, 9.21) | 12.54 (12.53, 12.56) | 3.67 (2.53, 4.83) |
| Latvia | 3.32 (3.31, 3.33) | 23.53 (23.50, 23.57) | 23.04 (20.62, 25.50) |
| Lithuania | 2.19 (2.18, 2.20) | 5.96 (5.94, 5.97) | 10.97 (9.01, 12.95) |
| Norway | 11.06 (11.04, 11.07) | 31.95 (31.92, 31.97) | 9.54 (7.11, 12.03) |
| Sweden | 12.07 (12.06, 12.09) | 44.35 (44.33, 44.38) | 15.56 (13.09, 18.09) |
| United Kingdom | 16.87 (16.86, 16.88) | 61.02 (61.01, 61.03) | 14.79 (12.90, 16.71) |
| **Europe (South)** | 7.27 (4.29, 12.33) | 4.89 (2.77, 8.65) | 2.68 (-0.36, 5.81) |
| Bosnia And Herzegovina | 0.00 (0.00, 0.00) | 3.65 (3.64, 3.66) | 31.17 (21.82, 41.24) |
| Croatia | 2.93 (2.92, 2.93) | 1.22 (1.21, 1.22) | -9.11 (-9.90, -8.32) |
| Greece | 8.33 (8.33, 8.34) | 9.89 (9.88, 9.90) | 2.69 (1.66, 3.74) |
| Italy | 5.71 (5.71, 5.71) | 5.73 (5.73, 5.74) | 0.38 (-0.21, 0.98) |
| Portugal | 13.1 (13.09, 13.11) | 14.81 (14.79, 14.82) | 2.14 (0.79, 3.51) |
| Serbia | 0.00 (0.00, 0.00) | 1.07 (1.06, 1.07) | 6.54 (2.72, 10.49) |
| Slovenia | 4.55 (4.53, 4.56) | 3.71 (3.70, 3.73) | -2.45 (-3.89, -0.98) |
| Spain | 17.86 (17.85, 17.87) | 22.22 (22.21, 22.23) | 2.12 (1.38, 2.87) |
| **Europe (East)** | 0.93 (0.36, 2.39) | 6.22 (3.12, 12.41) | 20.98 (16.42, 25.72) |
| Belarus | 0.05 (0.05, 0.05) | 1.20 (1.20, 1.21) | 34.36 (30.19, 38.67) |
| Bulgaria | 0.87 (0.86, 0.87) | 4.32 (4.31, 4.32) | 19.86 (12.73, 27.44) |
| Czech Republic | 7.15 (7.14, 7.16) | 20.99 (20.97, 21.00) | 11.29 (9.22, 13.40) |
| Hungary | 2.26 (2.25, 2.26) | 8.11 (8.10, 8.12) | 13.25 (10.84, 15.72) |
| Poland | 1.47 (1.47, 1.47) | 5.08 (5.08, 5.09) | 14.11 (12.19, 16.06) |
| Romania | 0.60 (0.60, 0.60) | 13.92 (13.91, 13.93) | 32.62 (23.02, 42.96) |
| Russia | 0.15 (0.15, 0.15) | 2.10 (2.10, 2.10) | 30.88 (22.32, 40.04) |
| Slovakia | 5.98 (5.97, 5.99) | 17.12 (17.10, 17.14) | 10.71 (8.56, 12.90) |
| **Oceania** | 8.60 (6.73, 11.00) | 16.72 (3.04, 92.01) | 6.87 (1.77, 12.23) |
| Australia | 7.59 (7.58, 7.59) | 7.00 (7.00, 7.01) | -2.55 (-4.82, -0.22) |
| New Zealand | 9.75 (9.73, 9.76) | 39.91 (39.88, 39.94) | 15.40 (14.48, 16.33) |
| **Asia (East)** | 0.57 (0.11, 3.07) | 1.60 (0.23, 11.04) | 10.86 (4.07, 18.08) |
| China | 0.01 (0.01, 0.01) | 0.33 (0.33, 0.33) | 41.59 (33.17, 50.53) |
| Japan | 1.02 (1.01, 1.02) | 0.83 (0.83, 0.83) | -5.57 (-9.12, -1.88) |
| South Korea | 6.43 (6.42, 6.43) | 9.31 (9.30, 9.31) | 4.28 (3.64, 4.93) |
| Taiwan | 1.75 (1.75, 1.75) | 2.59 (2.59, 2.59) | 3.15 (1.57, 4.75) |
| **Asia (Central)** | 0.00 (0.00, 0.00) | 0.26 (0.26, 0.26) | 4.17 (-0.96, 9.57) |
| Kazakhstan | 0.00 (0.00, 0.00) | 0.26 (0.26, 0.26) | 3.93 (-0.07, 8.08) |
| **Asia (West)** | 1.38 (0.37, 5.07) | 5.07 (2.36, 10.90) | 13.91 (5.99, 22.42) |
| Jordan | 0.75 (0.75, 0.76) | 3.33 (3.32, 3.33) | 6.55 (-1.69, 15.48) |
| Kuwait | 0.30 (0.29, 0.30) | 9.17 (9.15, 9.18) | 50.07 (32.06, 70.54) |
| Lebanon | 2.42 (2.41, 2.42) | 4.99 (4.98, 5.00) | 5.27 (2.14, 8.49) |
| Saudi Arabia | 1.06 (1.06, 1.06) | 7.81 (7.80, 7.81) | 20.88 (11.31, 31.27) |
| Türkiye | 11.82 (11.81, 11.82) | 25.18 (25.17, 25.18) | 7.12 (3.52, 10.84) |
| United Arab Emirates | 1.00 (1.00, 1.00) | 0.57 (0.56, 0.57) | -6.64 (-10.13, -3.01) |
| **Asia (South-east)** | 0.26 (0.26, 0.26) | 1.45 (0.07, 32.25) | 9.02 (1.04, 17.63) |
| Philippines | 0.26 (0.26, 0.26) | 0.30 (0.30, 0.30) | 0.13 (-1.66, 1.95) |
| Thailand | 0.00 (0.00, 0.00) | 7.06 (7.06, 7.07) | 17.48 (12.35, 22.85) |
| **Asia (South)** | 0.42 (0.25, 0.70) | 0.60 (0.18, 2.00) | 3.79 (-1.11, 8.94) |
| India | 0.32 (0.32, 0.32) | 1.11 (1.11, 1.11) | 14.01 (12.80, 15.24) |
| Pakistan | 0.54 (0.54, 0.54) | 0.33 (0.33, 0.33) | -4.58 (-5.95, -3.19) |
| **Africa (North)** | 0.33 (0.12, 0.88) | 0.86 (0.48, 1.54) | 17.46 (2.40, 34.73) |
| Algeria | 0.00 (0.00, 0.00) | 0.90 (0.90, 0.90) | 128.28 (-0.62, 424.40) |
| Egypt | 0.76 (0.76, 0.76) | 1.65 (1.65, 1.65) | 7.38 (5.61, 9.17) |
| Morocco | 0.07 (0.07, 0.07) | 0.40 (0.40, 0.40) | 26.94 (17.88, 36.70) |
| Tunisia | 0.65 (0.64, 0.65) | 0.91 (0.91, 0.91) | 2.59 (-0.83, 6.12) |
| **Africa (South)** | 0.52 (0.51, 0.52) | 0.66 (0.66, 0.66) | 3.41 (1.54, 5.31) |
| South Africa | 0.52 (0.51, 0.52) | 0.66 (0.66, 0.66) | 3.71 (2.39, 5.04) |

^a^Worldwide and regional estimates with 95% CI were calculated by pooling the estimates using meta-analysis (random-effects model).

^b^The average annual change is calculated using a linear regression model, with log-transformed consumption in DDD/TID as the dependent variable and year as the independent variable. The average annual change was expressed as average annual percentage change, calculated by [exp(the coefficient of the year variable) – 1] x 100%. The multinational and regional trend changes were estimated using linear mixed models, controlling for within-country correlations and assuming the correlations between years were autocorrelated.

Abbreviations: CI, confidence interval; DDD/TID, Defined Daily Dose per 10,000 inhabitants per day

# **Supplementary Table 7. Multinational, regional, and national levels of gabapentin enacarbil consumption in 2008 and 2018 and average annual percentage change.**

| **Year** | **United States DDD/TID (95%CI)**^a^ | **Puerto Rico DDD/TID (95%CI)**^a^ | **Japan**  **DDD/TID (95%CI)**^a^ | **Multinational DDD/TID (95%CI)**^a^ |
| --- | --- | --- | --- | --- |
| **2011** | 0.04 (0.04, 0.04) | - | - | **0.04** (0.0.4, 0.04) |
| **2012** | 0.13 (0.13, 0.13) | 0.00 (0.00, 0.00) | 0.09 (0.09, 0.09) | 0.02 (0.02, 0.03) |
| **2013** | 0.12 (0.12, 0.12) | 0.00 (0.00, 0.00) | 0.31 (0.31, 0.31) | 0.04 (0.02, 0.08) |
| **2014** | 0.23 (0.23, 0.23) | - | 0.44 (0.44, 0.44) | 0.31 (0.16, 0.61) |
| **2015** | 0.35 (0.35, 0.35) | 0.00 (0.00, 0.00) | 0.50 (0.50, 0.50) | 0.06 (0.05, 0.09) |
| **2016** | 0.51 (0.51, 0.51) | - | 0.54 (0.54, 0.54) | 0.52 (0.49, 0.56) |
| **2017** | 0.54 (0.54, 0.54) | - | 0.58 (0.58, 0.58) | 0.56 (0.52, 0.60) |
| **2018** | 0.47 (0.47, 0.47) | - | 0.63 (0.63, 0.64) | 0.55 (0.41, 0.73) |
| **Average annual percentage change (%, 95%CI)**^b^ | 41.44 (22.85, 62.85) | 5.93 (-96.85, 3467.65) | 29.13 (6.18, 57.04) | 35.86 (13.47, 62.66) |

^a^Worldwide and regional estimates with 95% CI were calculated by pooling the estimates using meta-analysis (random-effects model).

^b^The average annual change is calculated using a linear regression model, with log-transformed consumption in DDD/TID as the dependent variable and year as the independent variable. The average annual change was expressed as average annual percentage change, calculated by [exp(the coefficient of the year variable) – 1] x 100%. The multinational and regional trend changes were estimated using linear mixed models, controlling for within-country correlations and assuming the correlations between years were autocorrelated.

Abbreviations: CI, confidence interval; DDD/TID, Defined Daily Dose per 10,000 inhabitants per day

# **Supplementary Table 8. Multinational, regional, and national levels of pregabalin consumption in 2008 and 2018 and average annual percentage change.**

|  | **DDD/TID in 2008 (95%CI)^a^** | **DDD/TID in 2018 (95%CI)^a^** | **Average annual percentage change (%, 95%CI)^b^** |
| --- | --- | --- | --- |
| **Multinational** | 2.11 (1.56, 2.85) | 11.19 (8.71, 14.38) | 23.14 (20.07, 26.28) |
| **America (North)** | 18.93 (14.27, 25.12) | 39.11 (16.52, 92.59) | 7.53 (3.76, 11.43) |
| Canada | 16.39 (16.38, 16.39) | 60.71 (60.70, 60.72) | 13.86 (12.19, 15.55) |
| United States | 21.87 (21.87, 21.87) | 25.20 (25.19, 25.20) | 1.72 (0.98, 2.47) |
| **America (Central and Southern) and the Caribbean** | 1.30 (0.73, 2.31) | 5.31 (3.58, 7.88) | 17.78 (12.94, 22.82) |
| Argentina | 1.06 (1.06, 1.06) | 12.64 (12.64, 12.65) | 27.08 (18.25, 36.56) |
| Brazil | 0.00 (0.00, 0.00) | 5.12 (5.11, 5.12) | 39.23 (27.49, 52.05) |
| Chile | 1.69 (1.69, 1.69) | 9.07 (9.06, 9.07) | 20.04 (17.78, 22.34) |
| Colombia | 0.54 (0.53, 0.54) | 1.81 (1.81, 1.81) | 15.44 (12.45, 18.52) |
| Ecuador | 1.22 (1.22, 1.22) | 4.96 (4.96, 4.97) | 14.00 (10.65, 17.44) |
| Mexico | 2.08 (2.07, 2.08) | 3.12 (3.12, 3.12) | 3.76 (0.98, 6.63) |
| Peru | 0.25 (0.25, 0.26) | 1.86 (1.86, 1.86) | 20.75 (17.13, 24.48) |
| Puerto Rico | 12.63 (12.61, 12.65) | 11.99 (11.96, 12.01) | -0.79 (-1.32, -0.25) |
| Uruguay | 0.58 (0.58, 0.59) | 12.35 (12.33, 12.37) | 31.34 (19.72, 44.08) |
| Venezuela | 2.30 (2.29, 2.30) | 3.92 (3.92, 3.93) | 7.92 (0.26, 16.17) |
| **Europe (West)** | 14.06 (10.91, 18.12) | 39.87 (36.21, 43.90) | 11.00 (8.82, 13.23) |
| Austria | 7.54 (7.53, 7.55) | 39.87 (39.85, 39.90) | 17.39 (14.75, 20.09) |
| Belgium | 9.52 (9.51, 9.53) | 47.49 (47.47, 47.51) | 15.12 (11.01, 19.37) |
| France | 21.67 (21.67, 21.68) | 46.66 (46.65, 46.67) | 7.52 (6.06, 9.01) |
| Germany | 15.55 (15.55, 15.56) | 39.89 (39.88, 39.89) | 9.22 (7.90, 10.56) |
| Luxembourg | 23.96 (23.89, 24.03) | 37.80 (37.72, 37.89) | 4.49 (3.38, 5.62) |
| Netherlands | 0.00 (0.00, 0.00) | 32.41 (32.39, 32.42) | 7.74 (5.75, 9.77) |
| Switzerland | 13.32 (13.31, 13.34) | 37.11 (37.09, 37.13) | 10.71 (8.81, 12.65) |
| **Europe (North)** | 7.08 (5.03, 9.95) | 26.28 (18.36, 37.62) | 14.02 (10.08, 18.11) |
| Estonia | 1.27 (1.26, 1.28) | 17.17 (17.13, 17.21) | 29.30 (25.86, 32.83) |
| Finland | 33.96 (33.94, 33.99) | 51.85 (51.82, 51.89) | 3.09 (1.07, 5.15) |
| Ireland | 19.53 (19.51, 19.56) | 73.00 (72.96, 73.04) | 16.26 (11.80, 20.91) |
| Latvia | 0.84 (0.84, 0.85) | 2.86 (2.85, 2.87) | 9.04 (1.37, 17.30) |
| Lithuania | 1.28 (1.28, 1.29) | 14.16 (14.14, 14.18) | 26.21 (19.93, 32.82) |
| Norway | 23.24 (23.22, 23.27) | 34.37 (34.35, 34.40) | 4.97 (3.98, 5.96) |
| Sweden | 21.90 (21.89, 21.92) | 32.31 (32.29, 32.33) | 2.67 (0.63, 4.76) |
| United Kingdom | 13.59 (13.58, 13.59) | 77.86 (77.85, 77.87) | 19.16 (16.49, 21.89) |
| **Europe (South)** | 7.04 (4.88, 10.14) | 19.67 (14.06, 27.52) | 24.56 (17.40, 32.15) |
| Bosnia And Herzegovina | 0.00 (0.00, 0.00) | 1.68 (1.68, 1.69) | 60.01 (46.75, 74.46) |
| Croatia | 0.23 (0.23, 0.24) | 12.60 (12.58, 12.62) | 41.48 (29.16, 54.97) |
| Greece | 14.15 (14.14, 14.16) | 36.45 (36.43, 36.47) | 7.95 (6.02, 9.91) |
| Italy | 9.21 (9.20, 9.21) | 23.08 (23.08, 23.09) | 9.05 (7.23, 10.89) |
| Portugal | 18.13 (18.11, 18.14) | 36.72 (36.70, 36.74) | 6.59 (4.18, 9.06) |
| Serbia | 0.00 (0.00, 0.00) | 18.49 (18.48, 18.51) | 85.57 (63.59, 110.51) |
| Slovenia | 10.76 (10.74, 10.78) | 36.29 (36.24, 36.33) | 12.62 (10.94, 14.33) |
| Spain | 20.49 (20.48, 20.50) | 51.06 (51.05, 51.07) | 8.56 (6.06, 11.11) |
| **Europe (East)** | 0.49 (0.20, 1.23) | 7.47 (3.36, 16.62) | 31.18 (18.46, 45.26) |
| Belarus | 0.01 (0.01, 0.01) | 0.66 (0.66, 0.67) | 40.81 (28.01, 54.88) |
| Bulgaria | 0.19 (0.18, 0.19) | 9.45 (9.44, 9.47) | 30.46 (11.03, 53.28) |
| Czech Republic | 4.26 (4.26, 4.27) | 39.56 (39.54, 39.58) | 24.40 (22.09, 26.75) |
| Hungary | 4.95 (4.95, 4.96) | 13.40 (13.39, 13.41) | 10.47 (7.96, 13.03) |
| Poland | 0.04 (0.04, 0.04) | 11.71 (11.71, 11.72) | 74.65 (52.45, 100.09) |
| Romania | 2.51 (2.51, 2.52) | 2.45 (2.44, 2.45) | -5.22 (-10.13, -0.05) |
| Russia | 0.14 (0.14, 0.14) | 2.95 (2.95, 2.95) | 32.58 (6.40, 65.20) |
| Slovakia | 8.13 (8.12, 8.14) | 34.51 (34.48, 34.53) | 14.11 (10.18, 18.18) |
| **Oceania** | 0.79 (0.02, 32.51) | 30.08 (3.72, 243.26) | 39.97 (26.59, 54.76) |
| Australia | 5.28 (5.27, 5.28) | 87.38 (87.36, 87.40) | 38.82 (30.18, 48.03) |
| New Zealand | 0.12 (0.12, 0.12) | 10.35 (10.34, 10.37) | 34.69 (20.32, 50.79) |
| **Asia (East)** | 3.05 (3.05, 3.05) | 4.35 (0.89, 21.13) | 59.14 (25.27, 102.17) |
| China | 0.00 (0.00, 0.00) | 0.11 (0.11, 0.11) | 74.06 (31.12, 131.07) |
| Japan | 0.00 (0.00, 0.00) | 39.85 (39.85, 39.86) | 38.24 (13.43, 68.49) |
| South Korea | 3.05 (3.05, 3.05) | 21.69 (21.68, 21.69) | 19.61 (16.17, 23.14) |
| Taiwan | 0.00 (0.00, 0.00) | 3.83 (3.82, 3.83) | 66.52 (13.86, 143.54) |
| **Asia (Central)** | 0.00 (0.00, 0.00) | 0.78 (0.78, 0.79) | 34.97 (-1.41, 84.77) |
| Kazakhstan | 0.00 (0.00, 0.00) | 0.78 (0.78, 0.79) | 30.26 (16.88, 45.17) |
| **Asia (West)** | 1.41 (1.11, 1.79) | 9.20 (3.49, 24.22) | 21.00 (12.38, 30.28) |
| Jordan | 0.51 (0.51, 0.51) | 3.18 (3.18, 3.19) | 33.23 (20.26, 47.60) |
| Kuwait | 0.88 (0.87, 0.88) | 23.09 (23.06, 23.11) | 37.93 (31.50, 44.68) |
| Lebanon | 1.61 (1.60, 1.61) | 10.70 (10.69, 10.71) | 19.99 (16.62, 23.47) |
| Saudi Arabia | 2.56 (2.55, 2.56) | 3.55 (3.54, 3.55) | 12.15 (0.84, 24.73) |
| Türkiye | 2.05 (2.05, 2.05) | 35.81 (35.8, 35.81) | 34.95 (31.99, 37.98) |
| United Arab Emirates | 2.10 (2.09, 2.11) | 6.07 (6.07, 6.08) | 9.35 (0.72, 18.72) |
| **Asia (South-east)** | 0.27 (0.27, 0.27) | 1.72 (0.96, 3.10) | 15.78 (13.40, 18.21) |
| Philippines | 0.27 (0.27, 0.27) | 1.28 (1.27, 1.28) | 15.56 (14.41, 16.72) |
| Thailand | 0.00 (0.00, 0.00) | 2.32 (2.32, 2.33) | 13.80 (10.24, 17.49) |
| **Asia (South)** | 0.60 (0.31, 1.15) | 3.84 (3.51, 4.20) | 20.19 (13.42, 27.36) |
| India | 0.83 (0.83, 0.83) | 4.02 (4.02, 4.02) | 15.14 (12.04, 18.33) |
| Pakistan | 0.43 (0.43, 0.43) | 3.67 (3.66, 3.67) | 19.39 (14.34, 24.67) |
| **Africa (North)** | 0.38 (0.21, 0.68) | 13.56 (7.90, 23.28) | 42.98 (31.09, 55.95) |
| Algeria | 0.71 (0.71, 0.71) | 28.54 (28.53, 28.54) | 36.09 (26.77, 46.08) |
| Egypt | 0.31 (0.30, 0.31) | 37.90 (37.89, 37.90) | 53.80 (46.37, 61.60) |
| Morocco | 0.16 (0.16, 0.16) | 1.61 (1.61, 1.61) | 18.39 (12.75, 24.30) |
| Tunisia | 0.58 (0.58, 0.58) | 19.42 (19.41, 19.43) | 32.10 (25.13, 39.47) |
| **Africa (South)** | 0.64 (0.64, 0.65) | 3.08 (3.07, 3.08) | 16.93 (5.04, 30.17) |
| South Africa | 0.64 (0.64, 0.65) | 3.08 (3.07, 3.08) | 13.73 (9.81, 17.79) |

^a^Worldwide and regional estimates with 95% CI were calculated by pooling the estimates using meta-analysis (random-effects model).

^b^The average annual change is calculated using a linear regression model, with log-transformed consumption in DDD/TID as the dependent variable and year as the independent variable. The average annual change was expressed as average annual percentage change, calculated by [exp(the coefficient of the year variable) – 1] x 100%. The multinational and regional trend changes were estimated using linear mixed models, controlling for within-country correlations and assuming the correlations between years were autocorrelated.

Abbreviations: CI, confidence interval; DDD/TID, Defined Daily Dose per 10,000 inhabitants per day

**Supplementary Table 9. Healthcare systems classification of the 65 countries/ regions**

| **Country/ Region** | **Universal government-funded health system^a^** | **Universal public insurance system^b^** | **Universal public-private insurance^c^** | **Universal private health insurance system^d^** | **Non-universal insurance system^e^** |
| --- | --- | --- | --- | --- | --- |
| Canada^47^ | ✓ |  |  |  |  |
| United States^48^ |  |  |  |  | ✓ |
| Chile^49^ |  |  | ✓ |  |  |
| Puerto Rico^50^ |  |  |  |  | ✓ |
| Uruguay^51^ |  | ✓ |  |  |  |
| Austria^52^ |  | ✓ |  |  |  |
| Belgium^53^ |  | ✓ |  |  |  |
| France^54^ |  | ✓ |  |  |  |
| Germany^55^ |  |  | ✓ |  |  |
| Luxembourg^56^ |  | ✓ |  |  |  |
| Netherlands^57^ |  |  |  | ✓ |  |
| Switzerland^58^ |  |  |  | ✓ |  |
| Estonia^59^ |  | ✓ |  |  |  |
| Finland^60^ | ✓ |  |  |  |  |
| Ireland^61^ | ✓ |  |  |  |  |
| Latvia^62^ |  | ✓ |  |  |  |
| Lithuania^63^ |  | ✓ |  |  |  |
| Norway^64^ | ✓ |  |  |  |  |
| Sweden^65^ | ✓ |  |  |  |  |
| United Kingdom^66^ | ✓ |  |  |  |  |
| Czech Republic^67^ |  | ✓ |  |  |  |
| Hungary^68^ |  | ✓ |  |  |  |
| Poland^69^ |  | ✓ |  |  |  |
| Slovakia^70^ |  | ✓ |  |  |  |
| Croatia^71^ |  | ✓ |  |  |  |
| Greece^72^ |  | ✓ |  |  |  |
| Italy^73^ | ✓ |  |  |  |  |
| Portugal^74^ | ✓ |  |  |  |  |
| Slovenia^75^ |  | ✓ |  |  |  |
| Spain^76^ | ✓ |  |  |  |  |
| Australia^77^ | ✓ |  |  |  |  |
| New Zealand^78^ | ✓ |  |  |  |  |
| Japan^79^ |  | ✓ |  |  |  |
| South Korea^80^ |  | ✓ |  |  |  |
| Taiwan^81^ |  | ✓ |  |  |  |
| Kuwait^82^ | ✓ |  |  |  |  |
| Saudi Arabia^83^ | ✓ |  |  |  |  |
| United Arab Emirates^84^ |  |  |  | ✓ |  |
| Argentina^85^ |  |  | ✓ |  |  |
| Brazil^86^ | ✓ |  |  |  |  |
| Colombia^87^ |  | ✓ |  |  |  |
| Ecuador^88^ | ✓ |  |  |  |  |
| Mexico^89^ |  |  | ✓ |  |  |
| Peru^90^ |  |  | ✓ |  |  |
| Venezuela^91^ | ✓ |  |  |  |  |
| Belarus^92^ | ✓ |  |  |  |  |
| Bulgaria^93^ |  | ✓ |  |  |  |
| Romania^94^ |  | ✓ |  |  |  |
| Russia^95^ |  | ✓ |  |  |  |
| Bosnia and Herzegovina^96^ |  | ✓ |  |  |  |
| Serbia^97^ |  | ✓ |  |  |  |
| China^98^ |  | ✓ |  |  |  |
| Kazakhstan^99^ | ✓ |  |  |  |  |
| Jordan^100^ |  |  |  |  | ✓ |
| Lebanon^101^ |  |  |  |  | ✓ |
| Türkiye^102^ |  |  | ✓ |  |  |
| Thailand^103^ |  | ✓ |  |  |  |
| Algeria^104^ | ✓ |  |  |  |  |
| South Africa^105^ | ✓ |  |  |  |  |
| Philippines^106^ |  | ✓ |  |  |  |
| India^107^ | ✓ |  |  |  |  |
| Pakistan^108^ | ✓ |  |  |  |  |
| Egypt^109^ |  |  |  |  | ✓ |
| Morocco^110^ |  |  |  |  | ✓ |
| Tunisia^111^ |  |  |  |  | ✓ |

^a^Universal government-funded health system: The universal healthcare services in these countries are covered by taxation and healthcare services are available to all citizens regardless of their income or employment status.

^b^Universal public insurance system: Work force in these countries are asked to join some form of social insurance programmes. These programmes are sometimes also funded by the employers and the government. People who are unemployed or belongs to special patient groups may have their healthcare expenses covered by the government or become ineligible for free healthcare.

^c^Universal public-private insurance: Countries included will ask their citizens to enrol in private insurance plans. For those who are ineligible, other plans from the government will cover their healthcare expenses.

^d^Universal private health insurance system: Private healthcare insurance is mandatory in these countries. Citizens from low-income families will have government subsidised private insurance schemes.

^e^Non-universal insurance system: In these countries, not all citizens are insured. Some citizens may have private insurance coverage, while some others may have government subsidised public insurance plan. Patients who do not have insurance coverage will not be eligible for healthcare services.

**Supplementary Table 10. Reimbursement status of gabapentinoids in selected countries/ regions (as of 31^st^ May 2023)**

|  | **Reimbursement status of gabapentinoids** |
| --- | --- |
| Canada^112-115^ | Under the Canada Health Act, prescription drugs administered in Canadian hospitals are provided to patients at cost. Outside of hospital settings, provincial and territorial governments are responsible for administering their own publicly funded drug plans. These public drug plans determine which prescription drugs are covered and under what conditions for eligible recipients. Many Canadians and their family members have drug coverage through their employment, while others may have no effective drug coverage and must pay the full cost of prescription medications. The rates of co-payments for prescription drugs also vary among provinces.  For instance, in British Columbia, the Provincial and Territorial Public Drug Benefit Programme is called “Pharmacare”. It covers the cost of most drugs prescribed and the related dispensing fees with a maximum amount of CAD10. There are a total of 12 different plans under Pharamcare and they cover for different types of medications. However, eligibility of any of these plans will depend on the income level and medical conditions of the patient. One person can be covered by several plans.  Generic versions of gabapentin and pregabalin, disregarding their prescribed quantity and indications, are included into the drug list of Pharamcare and neither of them require pre-approval. However, brand product of pregabalin is not funded under the scheme. The maximum amount that Pharmacare covers will also depend on the brand and strength prescribed.  In Ontario, there are a total of 6 “Ontario Drug Benefit program” funded by the provincial government. The amount of deductible that needs to be paid by the patients will depend on their income and the type of programme they joined. Generic versions of gabapentin and pregabalin are included in the list of covered drugs of the programme. |
| United States^116-123^ | As of 2021, private health insurance is more commonly held than public coverage, with 66.0% of the population having private insurance and 35.7% having public coverage. Among the different types of health insurance, employer-based insurance is the most prevalent, covering 54.3% of the population, followed by Medicaid (18.9%), Medicare (18.4%), and direct-purchase coverage (10.2%).  As different private insurance plans vary in drug reimbursement coverage, we will focus on the two major public healthcare insurance programmes in the United States, which are Medicare and Medicaid.  Medicare is a federal health insurance program for individuals aged 65 or older. Individuals may also become eligible for Medicare if they have a disability, end-stage renal disease, or Lou Gehrig's disease. Part D of Medicare covers prescription drug reimbursement. Since 1^st^ January, 2006, everyone with Medicare, regardless of income, health status, or prescription drug usage has had access to prescription drug coverage. Monthly premiums for Part D are required. Monthly premiums are required for Part D, and the premium amount may change annually. Additionally, patients may have to pay an additional amount each month based on their income.  On the Medicare website, both gabapentin and pregabalin are included in the Part D drug plan formulary. The cost of the drugs paid by the patient will vary depending on the drug's "tier," the drug benefit phase the patient is in, the pharmacy used by the patient, and whether the patient receives "Extra Help" to cover Medicare drug coverage costs.  Medicaid is a joint federal and state program that provides health coverage to individuals with limited income and resources. As Medicaid is administered by individual states, co-payments, coinsurance, deductibles, and similar charges may be imposed on most Medicaid-covered benefits, including inpatient and outpatient services. The specific amounts of these charges vary based on income. Each state also maintains its own preferred drug list, which determines the amount charged for each prescription. For drugs on the preferred list, the maximum allowable co-payment is USD4 per item for all patients, regardless of income. For non-preferred drugs, patients with an income at or below 150% of the Federal Poverty Level have a maximum allowable co-payment of USD8, while those with higher income levels pay 20% of the agency's cost.  For instance, in New York state, the generic version of gabapentin and pregabalin are both listed as preferred drugs as anticonvulsants. Prior authorisation is required when they are used at a certain dose or concomitantly with opioids. Pregabalin solution and brand product of pregabalin are, however, listed as non-preferred drugs.  In contrast, the state of Mississippi has indicated a more expansive use for gabapentin and pregabalin as their generic versions are listed as preferred agents for fibromyalgia and neuropathic pain. Gabapentin is also listed as preferred anticonvulsant adjuvant. Certain brands are, however, listed as non-preferred agents. |
| Countries within European Medicine Agency^124-126^  (Austria, Belgium, Bulgaria, Croatia, Czech Republic, Estonia, Finland, France, Germany, Greece, Hungary, Ireland, Italy, Latvia, Lithuania, Luxembourg, Netherlands, Norway, Poland, Portugal, Romania, Slovakia, Slovenia, Spain and Sweden) | According to the “Medicines Reimbursement Policies in Europe” published by the World Health Organisation regional office for Europe in 2018, different European Union (EU) member states adopt various eligibility criteria for drug reimbursement coverage, and they depend on the medicine (product-specific) or the disease the medicine aims to treat (disease-specific). Reimbursement eligibility may also be linked to a specific population group in need of medicines (population groups-specific) or the total medicine expenditure of a patient within a certain period of time (consumption-based).  Product-specific reimbursement eligibility is determined based on factors such as therapeutic benefit, added value compared to alternatives, cost-effectiveness, and budget impact. Countries like the Netherlands, Italy, Czech Republic, and Slovakia primarily adopt this scheme.  Disease-specific reimbursement eligibility involves a list of specific diseases for which pharmaceutical treatment is reimbursed. The reimbursement rates for the same medicine may differ depending on the patient's disease. Estonia, Latvia, Lithuania, Ireland, France, Bulgaria, and Portugal use this scheme either as the main or supplementary approach.  Population groups-specific eligibility focuses on providing pharmaceutical reimbursement to specific groups in need, such as individuals requiring financial assistance due to their condition. Countries like Ireland, Finland, Hungary, Poland, and Romania combine this approach with other schemes.  Consumption-based reimbursement eligibility is based on the patient's pharmaceutical consumption within a specified period. Once a defined threshold of out-of-pocket payments is reached, the public payer covers additional pharmaceutical expenses during that period. Sweden primarily follows this scheme.  All included EU countries have reimbursement lists, with most applying a formulary indicating eligible drugs. Disease-specific reimbursement schemes often use a list of reimbursable diseases as the basis for coverage. Inclusion in an outpatient positive list doesn't guarantee full cost coverage; medicines may be partially reimbursed up to a specific percentage rate.  Austria, Croatia, Germany, Ireland, Italy, and the Netherlands provide 100% reimbursement for publicly subsidized medicines, but other payments like prescription charges, deductibles, or reference price system fees may still apply.  Most EU countries have a reference price system, where interchangeable medicines are grouped based on active substances or related subgroups. The public authority sets a reimbursed price for all medicines in the group. If the retail price exceeds the reference price, patients must pay the difference along with any applicable co-payments. The specifics of the reference price system vary among EMA countries.  We selected two EU member states as examples to investigate whether gabapentin and pregabalin are include in their drug reference list.  In Ireland, both gabapentin and pregabalin are included in the Health Service Executive Primary Care Reimbursement Service. In Germany, both medications are listed in the reference pricing lists of statutory health insurance funds. In Germany, both gabapentin and pregabalin are included in the reference pricing lists of the statutory health insurance fund. |
| United Kingdom^127-131^ | In the UK, the funding for individual drugs is determined by the local Integrated Care Boards (ICBs), formerly known as Clinical Commissioning Groups (CCGs). For example, both Gabapentin and Pregabalin are included in the North West London ICB's list of approved drugs.  In 2023, the prescription charge in England is £9.65 per item. Patients have the option to purchase an NHS Prescription Prepayment Certificate, which costs £31.25 for a three-month period or £111.60 for a 12-month period. However, certain patient groups, such as those under 16, those aged 60 or over, and patients with specific medical conditions, are exempt from prescription charges and receive their medications free of charge.  Prescriptions dispensed in Scotland under the NHS are free of charge. In NHS Wales, prescriptions are also provided free of charge. All prescriptions dispensed in Northern Ireland are free. |
| Australia^132,133^ | The Australian federal government supports inpatient and outpatient care through the Medicare Benefits Scheme (MBS) and helps with outpatient prescription medicines through the Pharmaceutical Benefits Scheme (PBS). The Department of Health is responsible for overseeing national policies and programs related to healthcare, including the MBS and PBS.  Medicare is the public health insurance system, and it is funded through national taxes, including a government levy. Private health insurance is widely accessible and offers coverage for out-of-pocket expenses and services from private healthcare providers. The government encourages individuals to enrol in private health insurance through tax rebates. The federal government subsidizes pharmaceuticals used in hospitals through the PBS. The cost-sharing for outpatient care may vary.  Starting from 1^st^ January 2023, patients may be required to pay up to AUD30.00 for most PBS medicines or AUD7.30 if they hold a concession card. The remaining cost, excluding brand premiums and certain charges, is covered by the Australian Government.  From 1^st^ January 2023, the PBS Safety Net threshold is set at AUD262.80 for patients with a concession card and AUD1,563.50 for other eligible patients. Once the Safety Net threshold is reached, general patients pay for further PBS prescriptions at the concessional co-payment rate, while concession card holders receive PBS prescriptions at no additional charge for the rest of the calendar year.  Some medicines may have a price premium or brand premium, which is an additional payment made by the patient to the supplier for a specified brand of a PBS medicine. If a patient opts for a more expensive brand, they are responsible for paying the price difference to the drug company, in addition to the co-payment.  The Pharmaceutical Benefits Scheme (PBS) maintains a schedule listing medicines available at government-subsidised prices. It is accessible to all Australian residents with a valid Medicare card.  Both gabapentin and pregabalin are included under the PBS schedule list. |
| New Zealand^134,135^ | All permanent residents in New Zealand have access to a comprehensive range of services that are primarily funded through pooled general taxes collected at the national level.  As stated in the manuscript, Pharmac is the body which decides which medications should be funded by the New Zealand government. It only decided to fund gabapentin throughout most of the studied period (2008-2018). Despite being a medication long registered in the market, it only decided to fund pregabalin in 2017.  Starting from 1^st^ July, 2023, patients will no longer be required to pay a standard NZD5 prescription charge for each prescription. Previously, a co-payment of NZD5 per prescription was applicable for the first 20 prescription items collected by a patient or their family within a year. However, prescriptions from specialists and non-publicly funded prescribers will incur a co-payment of NZD15. |
| China^136-138^ | According to the “2022 Drug Catalogue” issued by Ministry of Human Resources and Social Security, both gabapentin and pregabalin are included as Class B drugs in the formulary.  The "Class B List" is formulated by the state, and each province, autonomous region, and municipality directly under the Central Government may make appropriate adjustments on the list for not more than 15% of the total number of the drugs, according to the local economic level, medical needs, and prescribing habits.  Under the national medical insurance scheme, patients are required to contribute a certain percentage of the cost for Class B drugs. Furthermore, additional expenses may be incurred according to the provisions of the basic medical insurance. The specific proportion of self-payment is determined by the district and reported to the relevant Labour and Social Security administrative department at the provincial, autonomous region, or municipal level. The public insurance programs only reimburse patients up to a certain limit, beyond which individuals are responsible for covering all out-of-pocket costs. The ceiling for reimbursement varies based on factors such as the patient's occupation, place of residence, and other considerations. |
| Taiwan^81,139^ | In Taiwan, the National Health Insurance (NHI) program is mandatory for almost the entire population, including all nationals who have had a registered domicile in Taiwan for six months or more and all new-borns. The NHI premiums are jointly paid by the insured individuals, group insurance applicants, and the government.  The premium rates for NHI have undergone several adjustments over the years. From January 2016, the general premium rate was set at 4.69%, and supplementary premiums were introduced at a rate of 1.91%. The premiums payable by insured individuals depend on their category, and they are calculated based on the insured's premium rateable wage multiplied by the general premium rate.  NHI provides coverage for a wide range of healthcare services, including outpatient care, inpatient care, traditional Chinese medicine, dental care, child delivery, rehabilitation, home health care, and chronic mental illness rehabilitation. Medical payments under NHI cover various aspects such as diagnosis, examinations, laboratory tests, surgeries, anaesthesia, medications, materials, treatments, nursing, and insured beds.  In terms of pharmacy services, patients with a prescription from a contracted hospital or clinic can obtain medications at any contracted pharmacy. Co-payments are required for physician visits and prescription drugs, while coinsurance applies to inpatient care, subject to limits and exemptions. The co-payment for outpatient prescription drugs is capped at TWD200 per visit, regardless of the number of prescribed drugs. However, there is no annual cap on drug co-payments.  As of May 2023, only pregabalin is included in the drug list under the National Health Insurance, and there are specific restrictions on the insured indications for which it can be prescribed. |
| Japan^140-143^ | Japan’s statutory health insurance system (SHIS) is funded primarily by taxes and individual contributions. Enrollment in either an employment-based or residence-based health insurance plan is mandatory for all citizens. Benefits include hospital, primary, specialty, and mental health care, as well as prescription drugs. The system covers 98.3% of the population, with the remaining 1.7 percent covered by the Public Social Assistance Program. Health expenditures are funded by taxes (42%), mandatory individual contributions (42%), and out-of-pocket charges (14%). Contributions are shared between employers and employees in employment-based plans, while the national government, prefectures, and municipalities contribute to residence-based plans.  In employment-based health insurance plans, both employers and employees are required to contribute to the premiums. The contribution rates are typically around 10% of monthly salaries and bonuses and are based on the employee's income. There is a cap on the contribution amount. These contributions are tax-deductible and can vary depending on the type of insurance fund and the prefecture.  For residence-based health insurance plans, the national government, along with prefectures and municipalities, provides funding for a portion of individuals' mandatory contributions. It is worth noting that while more than 70% of the population holds some form of voluntary private health insurance, these private plans primarily serve as supplementary or complementary coverage alongside the statutory health insurance system.  All statutory health insurance plans in Japan offer the same benefits package, which includes hospital, primary, specialty, and mental health care, as well as prescription drugs. Enrolees typically pay a fixed percentage (usually 30%) as coinsurance or co-payment for health services and pharmaceuticals. However, the coinsurance rate may vary for certain special patient groups such as the elderly or children. The system also incorporates catastrophic coverage, with monthly out-of-pocket thresholds and an annual household out-of-pocket ceiling based on income and age.  In Japan, medication can be dispensed by both clinics and pharmacies. While doctors can directly provide medication to patients, the use of pharmacists has been growing, with the majority of prescriptions being filled at pharmacies.  The Central Social Insurance Medical Council plays a role in determining the SHIS list of covered pharmaceuticals and their prices. Price revisions for pharmaceuticals and medical devices are based on market surveys. The NHI drug price represents the final retail price of pharmaceuticals charged to patients and is set uniformly across the country.  Both Gabapentin and Pregabalin are included in the latest SHIS list in May 2023. |
| South Korea^144-147^ | The healthcare system in South Korea consists of two main components: mandatory social health insurance and medical aid. The National Health Insurance (NHI) system ensures healthcare coverage for all citizens, funded by contributions from the insured individuals and government subsidies. The medical aid program, on the other hand, provides healthcare services to low-income groups through government subsidies.  From 1^st^ January, 2022, the NHI premium rate, which includes long-term care insurance, is approximately 7.85% of monthly wages, with a cap on monthly contributions. The premium is split equally between employers and employees.  The Ministry of Health and Welfare (MoHW) is responsible for overseeing the National Health Insurance system, which includes two key institutions: the National Health Insurance Service (NHIS) and the Health Insurance Review & Assessment Service (HIRA). The NHIS serves as the insurer, while HIRA conducts reviews of claims and assesses the quality of healthcare services.  HIRA plays a role in determining the eligibility of products for reimbursement under the NHI system. The price recommended by HIRA sets a maximum reimbursement price for the drug, and when a product becomes off-patent and generics are available, the reimbursement price for the brand product is reduced.  The amount patients need to pay out-of-pocket, including medication fees, depends on the healthcare institute they visit. Visiting a tertiary general hospital usually results in higher out-of-pocket payments compared to clinics or other healthcare institutes. The out-of-pocket payment percentage may also vary for certain special patient groups.  Various formulations of Gabapentin and Pregabalin are eligible for reimbursement under the National Health Insurance scheme, and their respective maximum reimbursement prices are listed on the HIRA website. |

**Supplementary Table 11. Published guidelines related to the use of gabapentinoids between 2008-2018**

| **Country/ Region** | **Name of the guideline** | **Year** | **Issuing Body** | **Extracted context that is related to the use of gabapentinoids** |
| --- | --- | --- | --- | --- |
| United Kingdom | Generalised anxiety disorder and panic disorder in adults: management^148^ | 2011 | National Institute for Health and Care Excellence (NICE) | - If the person cannot tolerate selective serotonin reuptake inhibitor (SSRIs) or serotonin–norepinephrine reuptake inhibitor (SNRIs), consider offering pregabalin. |
|  | Epilepsies in children, young people and adults^149^ | 2012, update in 2022 | NICE | - Pregabalin is considered as a second-line add-on treatment option for focal seizures. - Gabapentin and pregabalin are included as medications that may exacerbate absence seizures. - Gabapentin and pregabalin are not recommended to use in myoclonic seizures as they may exacerbate seizures. - Gabapentin and pregabalin are included as medications that may exacerbate atonic seizures. - Gabapentin and pregabalin may exacerbate seizures in people with Dravet syndrome. - Gabapentin and pregabalin may exacerbate seizures in people with Lennox–Gastaut syndrome. - Gabapentin and pregabalin are included as medications that may exacerbate myoclonic-atonic seizures. |
|  | Neuropathic pain in adults: pharmacological management in non-specialist settings^150^ | 2013 | NICE | - All neuropathic pain (except trigeminal neuralgia) - Offer a choice of amitriptyline, duloxetine, gabapentin or pregabalin as initial treatment for neuropathic pain (except trigeminal neuralgia). See additional information for more on duloxetine, gabapentin and pregabalin. |
|  | Evidence-based pharmacological treatment of anxiety disorders, post-traumatic stress disorder and obsessive-compulsive disorder^151^ | 2014 | British Association for Psychopharmacology (BAP) | - Pregabalin is recommended as one of the treatment options for acute treatment of generalised anxiety disorder. - The dosage of pregabalin is also recommended to be increased if the initial treatments fail. - Pregabalin augmentation is recommended after a non-response to initial SSRI or SNRI treatment. |
|  | Restless legs syndrome: Oxycodone/naloxone prolonged release^152^ | 2015 | NICE | - Oxycodone/naloxone should only be considered after failure of dopaminergic therapy. Other non-dopaminergic drug treatment options for restless legs syndrome include off-label use of pregabalin, gabapentin, clonazepam or weak opioids such as codeine. - First-line drug treatment options for people with frequent or daily symptoms include non-ergot dopamine agonists (for example, pramipexole, ropinirole or rotigotine); pregabalin (off-label use) and gabapentin (off-label use). |
| Europe | EFNS guidelines on the pharmacological treatment of neuropathic pain: 2010 revision^153^ | 2010 | European Federation of Neurological Societies | - Both gabapentin and pregabalin are both listed as level A rating for treatment of neuropathic pain related to diabetic neuropathy and postherpetic neuralgia. - Pregabalin is listed as level A rating for treatment of central pain related neuropathic pain. |
| Canada | Canadian clinical practice guidelines for the management of anxiety, post-traumatic stress and obsessive-compulsive disorders^154^ | 2014 | Anxiety Disorders Association of Canada (ADAC) | - Gabapentin is included as third line treatment for panic disorder. - Pregabalin and Gabapentin are listed as first-line and second-line treatment option, respectively, for social anxiety disorder. - Pregabalin monotherapy is included as the first-line treatment option and as second-line adjunctive therapy option for generalised anxiety disorder. - Pregabalin is listed as third-line adjunctive therapy option for obsessive-compulsive disorder. - Pregabalin and Gabapentin are both listed as third-line adjunctive therapy option for posttraumatic stress disorder. |
|  | Pharmacological management of chronic neuropathic pain: Revised consensus statement from the Canadian Pain Society^155^ | 2014 | Canadian Pain Society | - Gabapentinoids (gabapentin and pregabalin) are recommended as first-line treatment options for neuropathic pain. |
| United States | Practice guideline for the treatment of Patients with Panic Disorder^156^ | 2009 | American Psychiatric Association (APA) | - After first- and second-line treatments and augmentation strategies have been exhausted (either due to lack of efficacy or intolerance of the treatment by the patient), less well-supported treatment strategies may be considered. These include monotherapy or augmentation with gabapentin or a second-generation antipsychotic or with a psychotherapeutic intervention other than Cognitive behavioural therapy (CBT) or Panic-Focused Psychodynamic Psychotherapy (PfPP). |
|  | Evidence-based guideline: Treatment of painful diabetic neuropathy^157^ | 2011 | American Academy of Neurology (AAN) | - Pregabalin is established as effective and should be offered for relief of painful diabetic neuropathy (PDN) (Level A). - Gabapentin is probably effective and should be considered for treatment of PDN (Level B). |
|  | Management of Postoperative Pain: A Clinical Practice Guideline^158^ | 2016 | the American Pain Society;  the American Society of Regional Anesthesia and Pain Medicine;  the American Society of Anesthesiologists’ Committee on Regional Anesthesia | - Gabapentin and pregabalin are included as medications that can be administered pre-operatively for Thoracotomy, Open laparotomy, Total hip replacement, Total knee replacement, Spinal fusion and Coronary artery bypass grafting. |
|  | Practice guideline update summary: Efficacy and tolerability of the new antiepileptic drugs I: Treatment of new-onset epilepsy^159^ | 2018 | American Academy of Neurology (AAN) | - Gabapentin may (Level C) be considered in decreasing seizure frequency in patients ≥60 years with new-onset focal epilepsy. - No high-quality studies suggest pregabalin is effective in treating new-onset epilepsy because no high-quality studies exist in adults of various ages. |
|  | Practice guideline update summary: Efficacy and tolerability of the new antiepileptic drugs II: Treatment-resistant epilepsy^160^ | 2018 | American Academy of Neurology (AAN) | - Immediate-release pregabalin (Level A) is established as effective to reduce seizure frequency for treatment-resistant adult focal epilepsy. |
| International | Guidelines for the pharmacological treatment of anxiety disorders, obsessive–compulsive disorder and posttraumatic stress disorder in primary care^161^ | 2012 | International Journal of Psychiatry in Clinical Practice | - Pregabalin is recommended as one of the first-line drugs for generalized anxiety disorder. |
|  | Pharmacotherapy for neuropathic pain in adults: systematic review, meta-analysis and updated NeuPSIG recommendations^162^ | 2015 | The Neuropathic Pain Special Interest Group (NeuPSIG) | - Both gabapentin and pregabalin are included as first-line options for treatment of neuropathic pain. |

**Supplementary References**

1 Health Canada. *Drug Product Database online query*, <<https://health-products.canada.ca/dpd-bdpp/dispatch-repartition>> (2023).

2 U.S. Food and Drug Administration. *FDA Label Search*, <<https://labels.fda.gov/getIngredientName.cfm>> (2023).

3 European Medicines Agency. *Neurontin - Article 30 referral - Annex I, II, III*, <<https://www.ema.europa.eu/en/documents/referral/neurontin-article-30-referral-annex-i-ii-iii_en.pdf>> (2023).

4 European Medicines Agency. *14/03/2023 Lyrica - EMEA/H/C/000546 - N/0122*, <<https://www.ema.europa.eu/en/documents/product-information/lyrica-epar-product-information_en.pdf>> (2023).

5 National Institute for Health and Care Excellence. *Gabapentin*, <<https://bnf.nice.org.uk/drugs/gabapentin/>> (2023).

6 National Institute for Health and Care Excellence. *Pregabalin*, <<https://bnf.nice.org.uk/drugs/gabapentin/>> (2023).

7 Therapeutic Goods Administration. *Product and Consumer Medicine Information*, <<https://www.ebs.tga.gov.au/ebs/picmi/picmirepository.nsf/PICMI?OpenForm&t=&q=gabapentin>> (2023).

8 Medsafe New Zealand Medicines and Medical Devices Safety Authority. *Data Sheets and Consumer Medicine Information*, <<https://www.medsafe.govt.nz/Medicines/SearchResult.asp>> (2023).

9 药智网. *加巴喷丁*, <<https://db.yaozh.com/pijian?name=%E5%8A%A0%E5%B7%B4%E5%96%B7%E4%B8%81>> (2023 ).

10 Pfizer Manufacturing Deutschland GmbH. *普瑞巴林胶囊说明书*, <<https://viatris.cn/-/media/project/viatris-sites/product-document/06pregabalin.pdf>> (2023).

11 Food and Drug Administration. *西藥、醫療器材、化粧品許可證查詢*, <<https://info.fda.gov.tw/>> (2022).

12 Pharmaceuticals and Medical Devices Agency. *Summary of investigation results Gabapentin, Gabapentinenacarbil*, <<https://www.pmda.go.jp/files/000211782.pdf>> (2016).

13 Pharmaceuticals and Medical Devices Agency. *Pregabalin*, <<https://www.pmda.go.jp/files/000197715.pdf#page=13>> (2014).

14 Health Insurance Review & Assessment Service. *보험인정기준*, <<https://www.hira.or.kr/rc/insu/insuadtcrtr/InsuAdtCrtrList.do?pgmid=HIRAA030069000400>> (2022).

15 Instituto de Salud Pública de Chile. *Consulta Productos Registrados*, <<https://registrosanitario.ispch.gob.cl/>> (2023).

16 Departamento de Salud. *Portal del Departamento de Salud*, <<https://www.salud.gov.pr/>> (2023).

17 Ministerio de Salud Pública. *Consulta de Medicamentos*, <<https://www.gub.uy/ministerio-salud-publica/home>> (2023).

18 Swissmedic. *Medicinal product information*, <<https://www.swissmedic.ch/swissmedic/en/home/services/medicinal-product-information.html>> (2019).

19 Ministry of health. *MOH Kuwait*, <<https://www.moh.gov.kw>> (2023).

20 Saudi Food and Drug Authority. *Drugs list*, <<https://www.sfda.gov.sa/en/drugs-list>> (2023).

21 Ministry of Health and Prevention. *Registered Medical Product Directory*, <<https://mohap.gov.ae/en/services/registered-medical-product-directory>> (2023).

22 National Administration of Drugs, F. a. M. D. *VADEMECUM NACIONAL DE MEDICAMENTOS*, <<http://anmatvademecum.servicios.pami.org.ar/index.html>> (2023).

23 ANVISA - AGÊNCIA NACIONAL DE VIGILÂNCIA SANITÁRIA. *Consultas*

<<https://consultas.anvisa.gov.br/>> (2023).

24 Instituto Nacional de Vigilancia de Medicamentos y Alimentos. *Alertas sanitarias medicamentos y productos biológicos*, <<https://www.invima.gov.co>> (2023).

25 Agencia Nacional de Regulación Control y Vigilancia Sanitaria. *Medication Consultation*, <<https://www.controlsanitario.gob.ec/>> (2023).

26 COFEPRIS - Mexico Ministry of Health. *Listados de Registros Sanitarios de Medicamentos*, <<https://www.gob.mx/cofepris/documentos/registros-sanitarios-medicamentos>> (2023).

27 Dirección General de Medicamentos Insumos y Drogas (DIGEMID). *Consulta de Registro Sanitario de Productos Farmacéuticos*, <<https://www.digemid.minsa.gob.pe/productospesquisados/principal/consultaproductospesquisados.aspx>> (2023).

28 Center for Expertise and Testing in Healthcare. *State Register of Medicinal Products of the Republic of Belarus*, <<https://www.rceth.by/Refbank>> (2023).

29 Ministry of Health of Russian Federation. *Ministry of Health of the Russian Federation*, <<https://minzdrav.gov.ru/>> (2023).

30 Agency for Medicinal Products and Medical Devices of Bosnia and Herzegovina. *Medicinal Products*, <<http://www.almbih.gov.ba/en/medicinal-products/>> (2023).

31 Medicines and Medical Devices Agency of Serbia. *Search of medicines and medical devices*, <<https://www.alims.gov.rs/english/medicinal-products/search-for-human-medicines/>> (2023).

32 Ministry of Health of the Republic of Kazakhstan. *National drug formulary of Kazakhstan*, <<https://www.knf.kz/kk>> (2023).

33 Jordan Food and Drug Administration. *Drug information, prices and leaflets*, <<http://www.jfda.jo/>> (2023).

34 Ministry of Public Health. *Lebanon National Drugs Database*, <<https://www.moph.gov.lb/en/Pages/3/3010/pharmaceuticals#/en/Drugs/index/3/3974/lebanon-national-drugs-database>> (2023).

35 Turkish Medicines and Medical Devices Agency. *List of Licensed Medicinal Products for Human Use*, <<https://www.titck.gov.tr>> (2023).

36 Food and Drug Administration. *Searching system for pharmaceutical products*, <<https://porta.fda.moph.go.th/>> (2023).

37 Ministry of Health, P. a. H. R. *Ministry of Health, Population and Hospital Reform*, <<http://www.sante.gov.dz>> (2023).

38 South African Health Products Regulatory Authority (SAHPRA) *Registered health products*, <<https://www.sahpra.org.za/databases-registers/>> (2023).

39 Food and Drug Administration. <<https://www.fda.gov.ph/>> (2023).

40 Central Drugs Standard Control Organization. *Drugs*, <<https://cdsco.gov.in/>> (2023).

41 Drug Regulatory Authority of Pakistan. *Registered drug index*, <<https://www.dra.gov.pk>> (2023).

42 Egyptian Drug Authority. *Egyptian Pharmacopoeia (content under construction)*, <<https://edaegypt.gov.eg/en/>> (2023).

43 Ministère de la Santé. *Ministère de la Santé*, <<https://www.sante.gov.ma/Pages/Accueil.aspx>> (2023).

44 Direction de la Pharmacie et du Médicament. *la liste des Médicaments ayant l'A.M.M.* , <<http://www.dpm.tn/>> (2023).

45 Royal Pharmaceutical Society of Great Britian. in *Martindale: the complete drug reference - Gabapentin* (Pharmaceutical Press, London, 2022).

46 Lexicomp. in *UpToDate - Gabapentin enacarbil: Drug information,* (Lexicomp, Inc., 2022).

47 Government of Canada. *Canada's Health Care System*, <<https://www.canada.ca/en/health-canada/services/health-care-system/reports-publications/health-care-system/canada.html>> (2019).

48 Roosa Tikkanen, R. O., Elias Mossialos, Ana Djordjevic, George A. Wharton. *International Health Care System Profiles - United States*, <<https://www.commonwealthfund.org/international-health-policy-center/countries/united-states>> (2020).

49 Bastias, G., Pantoja, T., Leisewitz, T. & Zarate, V. Health care reform in Chile. *CMAJ* **179**, 1289-1292 (2008). <https://doi.org:10.1503/cmaj.071843>

50 The Office of the Assistant Secretary for Planning and Evaluation (ASPE). (ed USA Department of Health & Human Services) (2017).

51 Pan American Health Organisation. Functioning of the health system in Uruguay - Principles, financing, management, and care model. (Pan American Health Organisation and Regional Office for the Americas - World Health Organization, 2020).

52 Bachner, F. *et al.* Austria: Health System Review. *Health Syst Transit* **20**, 1-254 (2018).

53 Gerkens, S. & Merkur, S. Belgium: Health System Review. *Health Syst Transit* **22**, 1-237 (2020).

54 Chevreul, K., Berg Brigham, K., Durand-Zaleski, I. & Hernandez-Quevedo, C. France: Health System Review. *Health Syst Transit* **17**, 1-218, xvii (2015).

55 Blumel, M., Spranger, A., Achstetter, K., Maresso, A. & Busse, R. Germany: Health System Review. *Health Syst Transit* **22**, 1-272 (2020).

56 OECD, Systems, E. O. o. H. & Policies. *Luxembourg: Country Health Profile 2021*. (2021).

57 Kroneman, M. *et al.* Netherlands: Health System Review. *Health Syst Transit* **18**, 1-240 (2016).

58 De Pietro, C. *et al.* Switzerland: Health System Review. *Health Syst Transit* **17**, 1-288, xix (2015).

59 Habicht, T. *et al.* Estonia: Health System Review. *Health Syst Transit* **20**, 1-189 (2018).

60 Keskimaki, I. *et al.* Finland: Health System Review. *Health Syst Transit* **21**, 1-166 (2019).

61 McDaid, D., Wiley, Miriam, Maresso, Anna. et al. Ireland: health system review. (2009).

62 Behmane, D. *et al.* Latvia: Health System Review. *Health Syst Transit* **21**, 1-165 (2019).

63 Murauskiene, L., Janoniene, R., Veniute, M., van Ginneken, E. & Karanikolos, M. Lithuania: health system review. *Health Syst Transit* **15**, 1-150 (2013).

64 Saunes, I. S., Karanikolos, M. & Sagan, A. Norway: Health System Review. *Health Syst Transit* **22**, 1-163 (2020).

65 Anell, A., Glenngard, A. H. & Merkur, S. Sweden health system review. *Health Syst Transit* **14**, 1-159 (2012).

66 Anderson, M. *et al.* United Kingdom: Health System Review. *Health Syst Transit* **24**, 1-194 (2022).

67 Bryndova, L. *et al.* Czechia: Health System Review. *Health Syst Transit* **25**, 1-216 (2023).

68 Gaal, P., Szigeti, S., Csere, M., Gaskins, M. & Panteli, D. Hungary health system review. *Health Syst Transit* **13**, 1-266 (2011).

69 Sowada, C. *et al.* Poland: Health System Review. *Health Syst Transit* **21**, 1-234 (2019).

70 Smatana, M. *et al.* Slovakia: Health System Review. *Health Syst Transit* **18**, 1-210 (2016).

71 Dzakula, A. *et al.* Croatia: Health System Review. *Health Syst Transit* **23**, 1-146 (2021).

72 Economou, C., Kaitelidou, D., Karanikolos, M. & Maresso, A. Greece: Health System Review. *Health Syst Transit* **19**, 1-166 (2017).

73 Giulio de Belvis, A. *et al.* Italy: Health System Review. *Health Syst Transit* **24**, 1-236 (2022).

74 de Almeida Simoes, J., Augusto, G. F., Fronteira, I. & Hernandez-Quevedo, C. Portugal: Health System Review. *Health Syst Transit* **19**, 1-184 (2017).

75 Albreht, T. *et al.* Slovenia: Health System Review. *Health Syst Transit* **23**, 1-183 (2021).

76 Bernal-Delgado, E. *et al.* Spain: Health System Review. *Health Syst Transit* **20**, 1-179 (2018).

77 Australian Government - Department of Health and Aged Care. *The Australian health system*, <<https://www.health.gov.au/about-us/the-australian-health-system>> (2019).

78 Jacqueline Cumming, J. M., Colin Barr, Greg Martin, Zac Gerring, Jacob Daubé. New Zealand health system review. *Health systems in transition* **Vol. 4** (2014).

79 Haruka Sakamoto, M. R., Shuhei Nomura, Etsuji Okamoto, Soichi Koike, Hideo Yasunaga, Norito Kawakami, Hideki Hashimoto, Naoki Kondo, Sarah Krull Abe, Matthew Palmer, Cyrus Ghaznavi. Japan health system review. *Health Systems in Transition* **Vol. 8** (2018).

80 Soonman Kwon, T.-j. L., Chang-yup Kim. Republic of Korea health system review. *Health systems in transition* **Vol. 5** (2015).

81 Cheng, T.-M. *International Health Care System Profiles - Taiwan*, <<https://www.commonwealthfund.org/international-health-policy-center/countries/taiwan>> (2020).

82 World Health Organization. Country Cooperation Strategy at a glance - Kuwait. (World Health Organization, 2017).

83 Alkhamis, A. Health care system in Saudi Arabia: an overview. *East Mediterr Health J* **18**, 1078-1079 (2012). <https://doi.org:10.26719/2012.18.10.1078>

84 Koornneef, E., Robben, P. & Blair, I. Progress and outcomes of health systems reform in the United Arab Emirates: a systematic review. *BMC Health Serv Res* **17**, 672 (2017). <https://doi.org:10.1186/s12913-017-2597-1>

85 Bello, M. & Becerril-Montekio, V. M. [The health system of Argentina]. *Salud Publica Mex* **53 Suppl 2**, s96-s108 (2011).

86 OECD. *OECD Reviews of Health Systems: Brazil 2021*. (2021).

87 OECD. *OECD Reviews of Health Systems: Colombia 2016*. (2015).

88 Flores Jimenez, S. E. & San Sebastian, M. Assessing the impact of the 2008 health reform in Ecuador on the performance of primary health care services: an interrupted time series analysis. *Int J Equity Health* **20**, 169 (2021). <https://doi.org:10.1186/s12939-021-01495-2>

89 Gonzalez Block, M. A., Reyes Morales, H., Hurtado, L. C., Balandran, A. & Mendez, E. Mexico: Health System Review. *Health Syst Transit* **22**, 1-222 (2020).

90 OECD. *OECD Reviews of Health Systems: Peru 2017*. (2017).

91 Page, K. R. *et al.* Venezuela's public health crisis: a regional emergency. *Lancet* **393**, 1254-1260 (2019). <https://doi.org:10.1016/S0140-6736(19)30344-7>

92 Richardson, E., Malakhova, I., Novik, I. & Famenka, A. Belarus: health system review. *Health Syst Transit* **15**, 1-118 (2013).

93 Dimova, A. *et al.* Bulgaria: Health System Review. *Health Syst Transit* **20**, 1-230 (2018).

94 Vladescu, C., Scintee, S. G., Olsavszky, V., Hernandez-Quevedo, C. & Sagan, A. Romania: Health System Review. *Health Syst Transit* **18**, 1-170 (2016).

95 Popovich, L. *et al.* Russian Federation. Health system review. *Health Syst Transit* **13**, 1-190, xiii-xiv (2011).

96 Juliane Winkelmann, Y. L., Boris Rebac. Health Systems in Action: Bosnia and Herzegovina. 28 (World Health Organization. Regional Office for Europe., 2022).

97 Bjegovic-Mikanovic, V. *et al.* Serbia: Health System Review. *Health Syst Transit* **21**, 1-211 (2019).

98 MENG Qingyue, Y. H., CHEN Wen, SUN Qiang, LIU Xiaoyun. People's Republic of China health system review. *Health systems in transition* **5** (2015).

99 OECD. *OECD Reviews of Health Systems: Kazakhstan 2018*. (2018).

100 Nazer, L. H. & Tuffaha, H. Health Care and Pharmacy Practice in Jordan. *Can J Hosp Pharm* **70**, 150-155 (2017). <https://doi.org:10.4212/cjhp.v70i2.1649>

101 Fleifel, M. & Abi Farraj, K. The Lebanese Healthcare Crisis: An Infinite Calamity. *Cureus* **14**, e25367 (2022). <https://doi.org:10.7759/cureus.25367>

102 Tatar, M. *et al.* Turkey. Health system review. *Health Syst Transit* **13**, 1-186, xiii-xiv (2011).

103 Pongpisut Jongudomsuk, S. S., Walaiporn Patcharanarumol, Supon Limwattananon, Supasit Pannarunothai, Patama Vapatanavong, Krisada Sawaengdee, Pinij Fahamnuaypol. The Kingdom of Thailand health system review. *Health systems in transition* **5** (2015).

104 Government of Canada. *Algeria health sector market profile*, <<https://www.tradecommissioner.gc.ca/algeria-algerie/market-reports-etudes-de-marches/0006431.aspx?lang=eng>> (2022).

105 Zwarenstein, M. The structure of South Africa's health service. *Afr Health*, 3-4 (1994).

106 Dayrit, M. M., Lagrada, L.P., Picazo, O.F., Pons, M.C. & Villaverde, M.C. The Philippines Health System Review. *Health Systems in Transition* **8** (2018).

107 Selvaraj S, K. K. A., Srivastava S, Bhan N, & Mukhopadhyay I. India health system review. *Health systems in transition* **11** (2022).

108 Kurji, Z., Premani, Z. S. & Mithani, Y. Analysis Of The Health Care System Of Pakistan: Lessons Learnt And Way Forward. *J Ayub Med Coll Abbottabad* **28**, 601-604 (2016).

109 Fasseeh, A. *et al.* Healthcare financing in Egypt: a systematic literature review. *J Egypt Public Health Assoc* **97**, 1 (2022). <https://doi.org:10.1186/s42506-021-00089-8>

110 Brown, D. B., Chibi, M. T., Hassani, N., Smith, S. C. & Searles, R. V. Moroccan Health Care: A Link to Radicalization and Proposed Solution. *Fed Pract* **36**, 510-513 (2019).

111 Chahed, M. K. & Arfa, C. Monitoring and evaluating progress towards Universal Health Coverage in Tunisia. *PLoS Med* **11**, e1001729 (2014). <https://doi.org:10.1371/journal.pmed.1001729>

112 Government of Canada. *Prescription drug insurance coverage*, <<https://www.canada.ca/en/health-canada/services/health-care-system/pharmaceuticals/access-insurance-coverage-prescription-medicines.html>> (2020).

113 The Government of British Columbia. *BC PharmaCare: Get help paying for medications and medical supplies*, <<https://www2.gov.bc.ca/gov/content/health/health-drug-coverage/pharmacare-for-bc-residents>> (2023).

114 Government of Ontario. *Get coverage for prescription drugs*, <<https://www.health.gov.on.ca/en/public/programs/drugs/>> (2023).

115 Government of Ontario. *Check medication coverage*, <<https://www.ontario.ca/check-medication-coverage/>> (2022).

116 Katherine Keisler-Starkey, L. N. B. Health Insurance Coverage in the United States: 2021. Report No. P60-278, (United States, 2022).

117 U.S. Department of Health and Human Services. *Who’s eligible for Medicare?*, <<https://www.hhs.gov/answers/medicare-and-medicaid/who-is-eligible-for-medicare/index.html>> (2022).

118 Medicare.gov. *Add prescription drug*, <<https://www.medicare.gov/plan-compare/#/manage-prescriptions?fips=06037&plan_type=&year=2023&lang=en>> (2023).

119 Medicaid.gov. *About Us*, <<https://www.medicaid.gov/about-us/index.html>> (2023).

120 Medicaid.gov. *Medicaid Drug Rebate Program (MDRP)*, <<https://www.medicaid.gov/medicaid/prescription-drugs/medicaid-drug-rebate-program/index.html#:~:text=The%20Medicaid%20Drug%20Rebate%20Program,drugs%20dispensed%20to%20Medicaid%20patients>> (2022).

121 Medicaid.gov. *Cost Sharing Out of Pocket Costs*, <<https://www.medicaid.gov/medicaid/cost-sharing/cost-sharing-out-pocket-costs/index.html>> (2023).

122 NYRx the New York Medicaid Pharmacy Program. *the Medicaid Pharmacy Program Preferred Drug List* <<https://newyork.fhsc.com/downloads/providers/NYRx_PDP_PDL.pdf>> (2023).

123 Mississippi Division of Medicaid. *Universal Preferred Drug List*, <<https://medicaid.ms.gov/preferred-drug-list/>> (2023).

124 World Health Organization. Regional Office for Europe. Medicines reimbursement policies in Europe. (World Health Organization. Regional Office for Europe., Denmark, 2018).

125 Health Service Executive (HSE), I. *Reimbursable Items*, <<https://www.sspcrs.ie/druglist/pub>> (2023).

126 Bundesinstitut für Arzneimittel und Medizinprodukte. *ABDA Festbetragsrecherche*, <<https://portal.dimdi.de/festbetragsrecherche/>> (2023).

127 North West London Integrated Care System. (ed United Kingdom National Health Service) (United Kingdom 2022).

128 NHS Business Services Authority United Kingdom. *NHS Prescription Prepayment Certificate (PPC)*, <<https://www.nhsbsa.nhs.uk/help-nhs-prescription-costs/nhs-prescription-prepayment-certificate-ppc>> (2023).

129 NHS inform Scotland. *Prescription charges and exemptions*, <<https://www.nhsinform.scot/care-support-and-rights/nhs-services/pharmacy/prescription-charges-and-exemptions>> (2023).

130 Welsh Government. *Free prescriptions*, <<https://www.gov.wales/free-prescriptions>> (2020).

131 Northern Ireland government. *Help with health costs*, <<https://www.nidirect.gov.uk/articles/help-health-costs#:~:text=All%20prescriptions%20dispensed%20in%20Northern,as%20everyone%20is%20automatically%20entitled>.> (2023).

132 Glover, L. *International Health Care System Profile - Australia*, <<https://www.commonwealthfund.org/international-health-policy-center/countries/australia#care-delivery-and-payment>> (2020).

133 Australian Government - Department of Health and Aged Care. *Pharmaceutical Benefits Scheme (PBS)*, <<https://www.pbs.gov.au/pbs/home>> (2023).

134 Ministry of Health New Zealand. *Prescription charges and the prescription subsidy scheme*, <<https://www.health.govt.nz/your-health/conditions-and-treatments/treatments-and-surgery/medications/prescription-charges-and-prescription-subsidy-scheme#:~:text=Many%20medicines%20in%20New%20Zealand,for%20prescriptions%20by%20approved%20providers>> (2023).

135 Pharmac New Zealand. *Community Schedule*, <<https://schedule.pharmac.govt.nz/ScheduleOnline.php>> (2023).

136 人力资源社会保障部, 国. *《国家基本医疗保险、工伤保险和生育保险药品目录（2022年）》的通知*, <<https://www.gov.cn/zhengce/zhengceku/2023-01/18/content_5737840.htm>> (2023).

137 中华人民共和国人力资源和社会保障部. *中华人民共和国社会保险法释义（十二）*, <<http://www.mohrss.gov.cn/fgs/syshehuibaoxianfa/201208/t20120807_28573.html>> (2012).

138 Fang, H. *International Health Care System Profile - China*, <<https://www.commonwealthfund.org/international-health-policy-center/countries/china>> (2020).

139 衛生福利部中央健康保險署. 2022-2023全民健康保險年報. (2022).

140 Matsuda, R. *International Health Care System Profiles - Japan*, <<https://www.commonwealthfund.org/international-health-policy-center/countries/japan>> (2020).

141 厚生労働省. *中央社会保険医療協議会 総会（第545回）議事次第*, <<https://www.mhlw.go.jp/stf/shingi2/0000212500_00186.html>> (2020).

142 Abe, Y. *Japan's NHI Drug Price System*, <<https://www.pmda.go.jp/files/000248690.pdf>> (2022).

143 Mahlich, J. & Sruamsiri, R. Co-insurance and health care utilization in Japanese patients with rheumatoid arthritis: a discontinuity regression approach. *Int J Equity Health* **18**, 22 (2019). <https://doi.org:10.1186/s12939-019-0920-7>

144 Health Insurance Review & Assessment Service. *Healthcare System in Korea*, <<https://www.hira.or.kr/dummy.do?pgmid=HIRAJ010000006000#:~:text=The%20health%20security%20system%20in,healthcare%20coverage%20to%20all%20citizens>> (

145 Park, J.-H. *Social security contributions*, <<https://taxsummaries.pwc.com/republic-of-korea/individual/other-taxes>> (2022).

146 Health Insurance Review & Assessment Service. *건강보험 본인부담기준 안내*, 2022).

147 Health Insurance Review & Assessment Service. *의약품안전사용서비스(DUR)*, <<https://www.hira.or.kr/ra/medi/form.do?pgmid=HIRAA030029000000&WT.gnb=%EC%9D%98%EC%95%BD%ED%92%88%EC%95%88%EC%A0%84%EC%82%AC%EC%9A%A9%EC%84%9C%EB%B9%84%EC%8A%A4%28DUR%29>> (

148 National Institute for Health and Care Exellence. Generalised anxiety disorder and panic disorder in adults: management. (2022).

149 National Institute for Health and Care Exellence. Epilepsies in children, young people and adults. (2022).

150 National Institute for Health and Care Exellence. Neuropathic pain in adults: pharmacological management in non-specialist settings. (2013).

151 Baldwin, D. S. *et al.* Evidence-based pharmacological treatment of anxiety disorders, post-traumatic stress disorder and obsessive-compulsive disorder: a revision of the 2005 guidelines from the British Association for Psychopharmacology. *J Psychopharmacol* **28**, 403-439 (2014). <https://doi.org:10.1177/0269881114525674>

152 National Institute for Health and Care Exellence. Restless legs syndrome: Oxycodone/naloxone prolonged release. (2015).

153 Attal, N. *et al.* EFNS guidelines on the pharmacological treatment of neuropathic pain: 2010 revision. *Eur J Neurol* **17**, 1113-e1188 (2010). <https://doi.org:10.1111/j.1468-1331.2010.02999.x>

154 Katzman, M. A. *et al.* Canadian clinical practice guidelines for the management of anxiety, posttraumatic stress and obsessive-compulsive disorders. *BMC Psychiatry* **14 Suppl 1**, S1 (2014). <https://doi.org:10.1186/1471-244X-14-S1-S1>

155 Moulin, D. *et al.* Pharmacological management of chronic neuropathic pain: revised consensus statement from the Canadian Pain Society. *Pain Res Manag* **19**, 328-335 (2014). <https://doi.org:10.1155/2014/754693>

156 Laura J. Fochtmann. PRACTICE GUIDELINE FOR THE Treatment of Patients With Panic Disorder. (American Psychiatric Association (APA), 2009).

157 Bril, V. *et al.* Evidence-based guideline: Treatment of painful diabetic neuropathy: report of the American Academy of Neurology, the American Association of Neuromuscular and Electrodiagnostic Medicine, and the American Academy of Physical Medicine and Rehabilitation. *Neurology* **76**, 1758-1765 (2011). <https://doi.org:10.1212/WNL.0b013e3182166ebe>

158 Chou, R. *et al.* Management of Postoperative Pain: A Clinical Practice Guideline From the American Pain Society, the American Society of Regional Anesthesia and Pain Medicine, and the American Society of Anesthesiologists' Committee on Regional Anesthesia, Executive Committee, and Administrative Council. *J Pain* **17**, 131-157 (2016). <https://doi.org:10.1016/j.jpain.2015.12.008>

159 Kanner, A. M. *et al.* Practice guideline update summary: Efficacy and tolerability of the new antiepileptic drugs I: Treatment of new-onset epilepsy: Report of the American Epilepsy Society and the Guideline Development, Dissemination, and Implementation Subcommittee of the American Academy of Neurology. *Epilepsy Curr* **18**, 260-268 (2018). <https://doi.org:10.5698/1535-7597.18.4.260>

160 Kanner, A. M. *et al.* Practice guideline update summary: Efficacy and tolerability of the new antiepileptic drugs II: Treatment-resistant epilepsy: Report of the American Epilepsy Society and the Guideline Development, Dissemination, and Implementation Subcommittee of the American Academy of Neurology. *Epilepsy Curr* **18**, 269-278 (2018). <https://doi.org:10.5698/1535-7597.18.4.269>

161 Bandelow, B. *et al.* Guidelines for the pharmacological treatment of anxiety disorders, obsessive-compulsive disorder and posttraumatic stress disorder in primary care. *Int J Psychiatry Clin Pract* **16**, 77-84 (2012). <https://doi.org:10.3109/13651501.2012.667114>

162 Finnerup, N. B. *et al.* Pharmacotherapy for neuropathic pain in adults: a systematic review and meta-analysis. *Lancet Neurol* **14**, 162-173 (2015). <https://doi.org:10.1016/S1474-4422(14)70251-0>
